# New Secondary Metabolites from Marine-Derived Fungus *Talaromyces minnesotensis* BTBU20220184

**DOI:** 10.3390/md22060237

**Published:** 2024-05-23

**Authors:** Weiliang Wang, Jingjing Wang, Fuhang Song, Renming Jia, Long Wang, Xiuli Xu, Na Yang

**Affiliations:** 1 Key Laboratory of Marine Mineral Resources and Polar Geology, Ministry of Education, School of Ocean Sciences, China University of Geosciences, Beijing 100083, China; wxw1134@126.com (W.W.); jingjingwang202404@163.com (J.W.); 2 Key Laboratory of Geriatric Nutrition and Health, Ministry of Education of China, School of Light Industry Science and Engineering, Beijing Technology and Business University, Beijing 100048, China; songfuhang@btbu.edu.cn; 3 Key Laboratory of Tropical Marine Ecosystem and Bioresource, Guangxi Key Laboratory of Beibu Gulf Marine Resources, Environment and Sustainable Development, Fourth Institute of Oceanography, Ministry of Natural Resources, Beihai 536000, China; jiarenming@4io.org.cn; 4 State Key Laboratory of Mycology, Institute of Microbiology, Chinese Academy of Sciences, Beijing 100101, China; wl_dgk@sina.com; 5 CAS Key Laboratory of Experimental Marine Biology, Center for Ocean Mega-Science, Institute of Oceanology, Chinese Academy of Sciences, Qingdao 266071, China; 6 Laboratory for Marine Biology and Biotechnology, Qingdao National Laboratory for Marine Science and Technology, Qingdao 266237, China

**Keywords:** marine-derived fungus, *Talaromyces minnesotensis*, antibacterial, *Staphylococcus aureus*

## Abstract

Six new compounds, talamitones A and B (**1** and **2**), demethyltalamitone B (**3**), talamiisocoumaringlycosides A and B (**4** and **5**), and talaminaphtholglycoside (**6**), together with six known compounds (**7**–**12**), were isolated from the marine-derived fungus *Talaromyces minnesotensis* BTBU20220184. The new structures were characterized by using HRESIMS and NMR. This is the first report of isocoumaringlycoside derivatives from a fungus of the *Talaromyces* genus. Compounds **5**, **6**, and **9** showed synergistic antibacterial activity against *Staphylococcus aureus*.

## 1. Introduction

The marine ecosystem is a rich pool for filamentous fungi. With the quick development in sampling using marine and molecular taxonomy technology, the fungal diversity of the marine environment has been greatly improved [1]. Over 1800 species of marine-derived fungi, belonging to 769 genera, have been studied, such as *Aspergillus*, *Penicillium*, *Trichoderma*, *Eurotium*, *Talaromyces*, and so on [2,3]. The section of *Talaromyces* was first introduced by Stolk and Samson in 1972 [4], and over 200 species have been reported in this genus [5,6]. Species of *Talaromyces* are commonly distributed in various environments, such as soil [6,7], marine [8], food products [9], leaf litter [10], and atmospheric environments [11]. A chemical investigation of *Talaromyces* species revealed the potential for new bioactive chemical entries [12]. A panel of new structural classes have been reported from this fungus, including ergosteroids [13,14], meroterpenoids [15], and pyridone derivatives [16,17]. Nicoletti reviewed the chemical diversity of *Talaromyces* species from marine environments. Over 500 natural products have been identified from fungi of the *Talaromyces* genus, and 45% of the compounds were new structures. These compounds displayed a wide spectrum of biological activities and chemical diversity, attracting researchers to screening new pharmaceutical entries [18].

**Figure 1:**
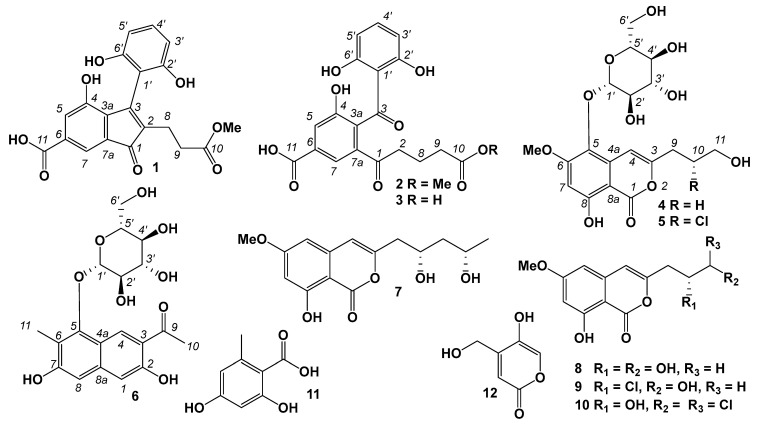
Chemical structures of **1**–**12**.

In our ongoing chemical investigation of marine-derived microorganisms, new antibacterial compounds have been identified from marine-derived *Talaromyces* strains [19,20]. Further research on a marine-derived fungus from Qinzhou Bay, Guangxi Province, China, resulted in the identification of twelve compounds, including six new compounds, talamitones A and B (**1** and **2**), demethyltalamitone B (**3**), talamiisocoumaringlycosides A and B (**4** and **5**), and talaminaphtholglycoside (**6**), along with six reported compounds, mucorisocoumarin B (**7**), diaportinol (**8**), peniisocoumarin E (**9**), dichlorodiaportin (**10**), orsellinic acid (**11**), and 4-(hydroxymethyl)-5-hydroxy-2*H*-pyran-2-one (**12**) (Figure 1), from *T*. *minnesotensis* BTBT20220184. Compounds **5**, **6,** and **9** showed synergistic antibacterial activity against *Staphylococcus aureus*. This paper focuses on the isolation, detailed structure elucidation, and antimicrobial activity evaluation of these compounds.

## 2. Results

### 2.1. Structure Determination

**Figure 2:**
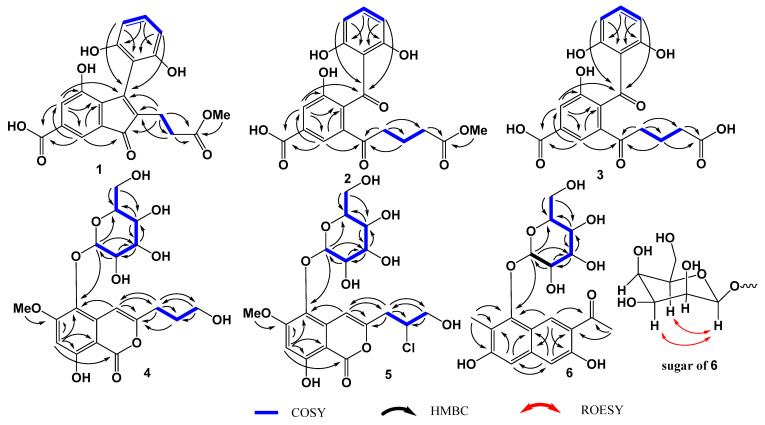
Key COSY (bold lines) and HMBC (arrows) correlations in **1**–**6**.

Compound **1** was isolated as a light-yellow oil. The molecular formula of **1** was determined to be C_20_H_16_O_8_ based on the HRESIMS spectrum (*m/z* [M + H]^+^ 385.0923, calcd for C_20_H_17_O_8_, 385.0918), indicating thirteen degrees of unsaturation (Figure S1). The ^1^H NMR data of **1** (Table 1, Figure S2) showed five aromatic protons at *δ*_H_ 7.50 (d, *J* = 1.5 Hz, H-5), 7.51 (d, *J* = 1.5 Hz, H-7), 6.39 (d, *J* = 8.0 Hz, H-3′, 5′), and 7.04 (t, *J* = 8.0 Hz, H-4′); two methylenes at *δ*_H_ 2.44 (m, H_2_-8) and 2.46 (m, H_2_-9); and one methoxy at *δ*_H_ 3.60 (s, 10-OCH_3_). The ^13^C and HSQC spectra of **1** (Figures S3 and S4) displayed twenty carbon signals (Table 1), including one carbonyl at *δ*_C_ 198.5 (C-1); two carboxyl carbons at *δ*_C_ (175.3, C-10) and (168.9, C-11); eight sp^2^ quaternary carbons at *δ*_C_ 136.7 (C-2), 154.0 (C-3), 134.8 (C-3a), 152.6 (C-4), 134.4 (C-6), 133.1 (C-7a), 110.7 (C-1′), and 156.7 (C-2′, 6′); five sp^2^ methine carbons at *δ*_C_ 126.8 (C-5), 115.4 (C-7), 107.6 (C-3′, 5′), and 130.9 (C-4′); two methylene carbons at *δ*_C_ 20.7 (C-8) and 32.9 (C-9); and one methoxy carbon at *δ*_C_ (52.1, 10-OCH_3_). The ^1^H-^1^H COSY correlations (Figure 2 and Figure S5) revealed the fragments of C-8/C-9 and C-3′/C-4′/C-5′. In the HMBC spectrum (Figure S6), the correlations from H-5 to C-3, C-3a, C-4, C-6, and C-7; from H-7 to C-1, C-3a, and C-5; and from H-5 and H-7 to C-11, along with the chemical shift of C-4 (*δ*_C_ 152.6), suggested that the hydroxyl and carboxyl substituted the inden-1-one moiety (Figure 2). The HMBC correlations from H-4′ to C-2′ and C-6′ and from H-3′ and H-5′ to C-1′ indicated the 1,2,3-trisubstituted benzene fragment. The HMBC correlations from H_2_-8, H_2_-9, and H_3_-OMe to C-10 confirmed the presence of a C-8/C-9/C-10/OMe moiety. The HMBC correlations from H_2_-8 to C-1, C-2, and C-3; from H_2_-9 to C-2; and from H-3′ and H-5′ to C-1′ and C-3 revealed the connection from C-2 to C-8 and from C-3 to C-1′, respectively. Therefore, the structure of **1** was assigned as shown in Figure 1 and named talamitone A.

Compound **2** was isolated as a light-yellow powder. The molecular formula of **2** was determined to be C_20_H_18_O_9_ based on the HRESIMS spectrum (*m/z* [M + H]^+^ 403.1011, calcd for C_20_H_19_O_9_, 403.1024), indicating twelve degrees of unsaturation (Figure S7). The analysis of the ^1^H, ^13^C, HSQC, and ^1^H-^1^H COSY spectra (Table 1, Figures S8–S11) revealed the presence of two substituted benzene rings (*δ*_C_ 136.8, C-3a; *δ*_C_ 155.2, C-4; *δ*_C_ 121.7/*δ*_H_ 7.68, C-5/H-5; *δ*_C_ 132.8, C-6; *δ*_C_ 122.0/*δ*_H_ 8.07, C-7/H-7; *δ*_C_ 137.3, C-7a; *δ*_C_ 112.2, C-1′; *δ*_C_ 163.3, C-2′,6′; *δ*_C_ 108.1/*δ*_H_ 6.28, C-3′,5′/H-3′,5′; *δ*_C_ 137.4/*δ*_H_ 7.21, C-4′/H-4′); three sp^3^ methylenes (*δ*_C_ 38.8/*δ*_H_ 3.06, C-2/H-2; *δ*_C_ 20.4/1.90, C-8/H-8; *δ*_C_ 33.7/*δ*_H_ 2.35, C-9/H-9); one methoxy (*δ*_C_ 52.1/*δ*_H_ 3.64, 10-OCH_3_); two carbonyls at *δ*_C_ 201.0 (C-1) and 202.8 (C-3); as well as two carboxyls at *δ*_C_ 175.5 (C-10) and *δ*_C_ 168.6 (C-11). All of the above NMR data indicated that the structure of **2** is similar to that of **1**. A detailed comparison of the NMR data of compounds **1** and **2** showed that the double bond C-2/C-3 was modified. The double bonds of C-2/C-3 in **1** were replaced by one sp^3^ methylene (*δ*_H_ 3.06, 2H, t, *J* = 7.0 Hz; *δ*_C_ 38.8, C-2) and one carbonyl (*δ*_C_ 202.8, C-3). In the HMBC spectrum (Figure 2 and Figure S12), the long-range correlation from H-5 to C-3 confirmed the carbonyl (C-3)’s bearing on C-3a. The HMBC correlations from H-3′ and H-5′ to C-3 and from H_2_-2 to C-1 and C-7a indicated that the α,β-unsaturated moiety of C-1/C-2/C-3 in **1** changed to the moiety of C-1 (carbonyl)/C-2 (methylene) and the other carbonyl of C-3 in **2**. Therefore, the structure of **2** was assigned as shown in Figure 1 and named talamitone **B**.

Compound **3** was isolated as a light-yellow powder. The molecular formula of **3** was determined to be C_19_H_16_O_9_ based on the HRESIMS spectrum (*m/z* [M + H]^+^ 389.0871, calcd for C_19_H_17_O_9_, 389.0867), indicating twelve degrees of unsaturation (Figure S13). Compound **3** showed similar NMR data to those of **2**. By comparing the molecular formula and NMR data (Table 1, Figures S14 and S15), **3** was deduced to be the product of the demethylation of **2**. The structure of **3** was confirmed by detailed analysis of the HSQC, ^1^H-^1^H COSY, and HMBC spectra (Figures S16–S18). The long-range correlation from H-5 to C-3 confirmed the carbonyl (C-3)’s bearing on C-3a. The presence of HMBC correlations from H_2_-8 and H_2_-9 to C-10 indicated the carboxyl of C-10. Therefore, the structure of **3** was assigned as shown in Figure 1 and named demethyltalamitone **B**.

Compound **4** was isolated as a light-yellow powder. The molecular formula of **4** was determined to be C_19_H_24_O_11_ based on the HRESIMS spectrum (*m/z* [M + Na]^+^ 451.1204, calcd for C_19_H_24_O_11_Na, 451.1211), indicating eight degrees of unsaturation (Figure S19). The ^1^H and ^13^C NMR data of **4** (Table 2, Figures S20 and S21) showed signals for one isocoumarin substructure at *δ*_C_ 167.9 (C-1, carboxyl), 157.9 (C-3, oxygenated), *δ*_C_ 101.2 (C-4)/*δ*_H_ 6.94 (s, H-4), 133.5 (C-4a), 132.9 (C-5), 161.1 (C-6, oxygenated), *δ*_C_ 99.7 (C-7)/*δ*_H_ 6.63 (s, H-7), 161.8 (C-8, oxygenated), and 99.3 (C-8a); one hydroxypropyl at *δ*_C_ 30.7 (C-9)/*δ*_H_ 2.63 (m, H_2_-9), *δ*_C_ 30.8 (C-10)/*δ*_H_ 1.90 (tt, *J* = 7.0 Hz, H_2_-10), and *δ*_C_ 61.7, C-11/*δ*_H_ 3.63 (m, H_2_-11); one glucose at *δ*_C_ 105.9 (C-1′)/*δ*_H_ 4.70 (d, *J* = 7.5 Hz, H-1′), *δ*_C_ 75.6 (C-2′)/ *δ*_H_ 3.50 (m, H-2′), *δ*_C_ 77.8 (C-3′)/*δ*_H_ 3.42 (m, H-3′), *δ*_C_ 71.2 (C-4′)/*δ*_H_ 3.42 (m, H-4′), *δ*_C_ 78.2, C-5′/*δ*_H_ 3.17 (m, H-5′), *δ*_C_ 62.5, C-6′)/ *δ*_H_ 3.76 (dd, *J* = 11.5, 2.5 Hz, H-6′a), and 3.67 1H (dd, *J* = 11.5, 4.5 Hz, H-6′b); and one methoxy at *δ*_C_ 56.8/*δ*_H_ 3.94 (s, 6-OCH_3_). The protonated carbons and correlations were confirmed by the HSQC and ^1^H-^1^H COSY spectra (Figures S22 and S23). In the HMBC spectrum (Figure 2 and Figure S24), the correlations from H_3_-6-OMe to C-6 revealed the methoxy’s bearing on C-6. The HMBC correlations from H-4 to C-3 and C-9 and from H_2_-9 and H_2_-10 to C-3 indicated the hydroxypropyl at C-3. The connection between C-5 and C-1′ through an oxygen atom was confirmed by the HMBC correlations from H-1′ to C-5. Therefore, the planar structure of **4** was assigned and named talamiisocoumaringlycoside A.

Compound **5** was isolated as a light-yellow powder. The molecular formula of **5** was determined to be C_19_H_23_ClO_11_ based on the HRESIMS spectrum (*m/z* [M + Na]^+^ 485.0830, calcd for C_19_H_23_ClO_11_Na, 485.0821), indicating eight degrees of unsaturation (Figure S25). The ^1^H and ^13^C NMR data of **5** (Table 2, Figures S26 and S27) showed similar signals to those of **4**, except for the methylene of CH_2_-10, which was replaced by one sp^3^ methine. Through analysis of the molecular formula, chemical shift of ^13^C NMR, and 2D NMR data (Figures S28–S30), the chloro-substituted methine at *δ*_C_ 60.4 (C-4)/*δ*_H_ 4.32 (m, H-4) was deduced. By comparison of the optical rotations of **5** (${\left[\alpha \right]}_{D}^{25}$ + 26.0) and peniisocoumarin E (${\left[\alpha \right]}_{D}^{25}$ + 69.2), the configuration of C-10 was assigned to be the same as the known compound peniisocoumarin E (**9**) [21]. Therefore, the planar structure of **5** was assigned as shown in Figure 1 and named talamiisocoumaringlycoside **B**.

Compound **6** was isolated as a light-yellow powder. The molecular formula of **6** was determined to be C_19_H_22_O_9_ based on the HRESIMS spectrum (*m/z* [M + H]^+^ 395.1338, calcd for C_19_H_23_O_9_, 395.1337), indicating nine degrees of unsaturation (Figure S31). The ^1^H and ^13^C NMR data of **5** (Table 2, Figures S32 and S33) showed signals for one glucose moiety as in **4** or **5** at *δ*_C_ 107.1 (C-1′)/*δ*_H_ 4.78 (d, *J* = 7.5 Hz, H-1′), 75.8 (C-2′)/*δ*_H_ 3.69 (m, H-2′), 78.2 (C-3’)/*δ*_H_ 3.47 (m, H-13′), 71.4 (C-4′)/*δ*_H_ 3.47 (m, H-4’), 77.9 (C-5′)/*δ*_H_ 3.13 (m, H-5’), *δ*_C_ (62.6, C-6’)/*δ*_H_ 3.76 (dd, *J* = 2.5, 11.5 Hz, H-6’a), and 3.67 (m, H-6’b); three aromatic methines at *δ*_C_ 110.0 (C-1)/*δ*_H_ 6.89 (s, H-1), 131.3 (C-4)/*δ*_H_ 9.17 (s, H-4), and 104.4 (C-8)/*δ*_H_ 6.76 (s, H-8); two methyls at *δ*_C_ 27.2 (C-10)/*δ*_H_ 2.76 (s, H_3_-10) and 10.9 (C-11)/*δ*_H_ 2.34 (s, H_3_-11); one carbonyl at *δ*_C_ 207.0 (C-9); and seven sp^2^ quaternary carbons at *δ*_C_ 158.4 (C-2, oxygenated), 119.5 (C-3), 118.6 (C-4a), 154.0 (C-5, oxygenated), 120.7 (C-6), 160.5 (C-7, oxygenated), and 140.4 (C-8a). The protonated carbons were confirmed by the HSQC and ^1^H-^1^H COSY spectra (Figures S34 and S35). The substituted naphthalene substructure was confirmed by the HMBC correlations (Figure 2 and Figure S36) from H-1 to C-3, C-4a, and C-8; from H-4 to C-2, C-5, C-8a, and C-9; from H-8 to C-1, C-4a, and C-6; from H_3_-10 to C-3 and C-9; as well as from H_3_-11 to C-5, C-6, and C-7. The connection between C-5 and C-1′ through an oxygen atom was characterized by the HMBC correlation from H-1′ to C-5. Therefore, the planar structure of **6** was assigned and named talaminaphtholglycoside.

With almost identical NMR data, the sugar moieties of **4**–**6** were deduced to be the same configurations. The anomeric protons H-1′ of **4**–**6** showed large coupling constants (*J* = 7.5 Hz), indicating the presence of a β-glycosidic bond [22]. In the ROESY spectrum (Figure 2 and Figure S37), H-1′ showed crossing peaks with H-3′ and H-5′, which revealed the axial–axial relationship. Through a detailed comparison of the ^13^C NMR data for the sugar moiety of 1-hydroxy-3-methoxy-8-methyl-2-*O*-β-D-glucopyranosylnaphthaline [23] with those of **4**–**6**, the sugar moieties were determined as β-D glucopyranose.

The known compounds were isolated from *T*. *minnesotensis* BTBT20220184 and determined as mucorisocoumarin B (**7**) [24], diaportinol (**8**) [25], peniisocoumarin E (**9**) [21], dichlorodiaportin (**10**) [25], orsellinic acid (**11**) [26], and 4-(hydroxymethyl)-5-hydroxy-2*H*-pyran-2-one (**12**) [27] by comparing their spectroscopic data with those reported in the literature.

### 2.2. Biological Activity

The biological evaluations were conducted to assess the antibacterial activities against *Candida albicans* ATCC 10231, *Staphylococcus aureus* ATCC 25923, and *Escherichia coli* ATCC 25923. None of the compounds showed markable antimicrobial activities, except for **8** inhabiting the growth of *S. aureus* at 200 μg/mL. When combing them with 0.125 μg/mL of methicillin, **5**, **6,** and **9** showed synergistic antibacterial activity against *S. aureus* at concentrations of 25, 50, and 12.5 μg/mL, respectively.

## 3. Materials and Methods

### 3.1. General Experimental Procedures

Optical rotations ${\left[\alpha \right]}_{D}^{25}$ were recorded on an Anton Paar MCP 200 Modular Circular Polarimeter (Austria) in a 100 × 2 mm cell. NMR spectra were measured on a Bruker Avance 500 spectrometer with residual solvent peaks as references (CD_3_OD: *δ*_H_ 3.31, *δ*_C_ 49.0). High-resolution HRESIMS measurements were obtained on a Xevo G2 XS QToF system (Manchester, UK) in positive-ion mode. HPLC was performed using an Agilent 1200 Series separation module equipped with an Agilent 1200 Series diode array and Agilent 1260 Series fraction collector and an Agilent ZORBAX RX-C8 column (250 × 9.4 mm, 5 µm).

### 3.2. Microbial Material, Fermentation, Extraction, and Purification

The strain *T*. *minnesotensis* BTBU20220184 was isolated from a sediment sample collected from Qinzhou Bay, Guangxi Province, China. It was cultured on a malt extract agar plate at 26 °C for 4 days. The genomic DNA of *T*. *minnesotensis* BTBU20220184 were extracted using a Fungi Genomic DNA Extraction Kit (Solarbio Life Sciences, Beijing, China). The β-tubulin gene sequence region was amplified using the conventional primer pair of Bt2a (5′-GGTAACCAAATCGGTGCTGCTTTC-3′) and Bt2b (5′-ACCCTCAGTGTAGTGACCCTTGGC-3′). PCR products were sent to Beijing Qingke Biotechnology Co., Ltd. (Beijing, China) for DNA sequencing. BTBU20220184 was identified as *T*. *minnesotensis* by comparing the β-tubulin gene sequence with the GenBank database using the BLAST program. A neighbor-joining (NJ) tree (Figure S38) was constructed using a software package, Mega version 5 [28]. The fungus was assigned the accession number BTBU20220184 in the culture collection at Beijing Technology and Business University, Beijing.

The strain *T*. *minnesotensis* BTBU20220184 was inoculated on a potato dextrose agar plate and cultured for 5 days. Then, a slit of agar with fungus was cut from the plate and inoculated into 250 mL conical flasks as seeds and cultured at 28 °C (180 rpm) for 3 days. Then, 5 mL of seed culture was added in to twenty 1 L conical flasks, each containing a solid medium consisting of rice (160 g) and artificial seawater (3.5%; 140 mL), and the flasks were incubated under static conditions at 26 °C for 29 days. The cultures were extracted three times with a mixture of EtOAc:MeOH (80:20), and the combined extracts were evaporated in vacuo to yield a residue. The residue was suspended in 800 mL distilled water and partitioned with isometric EtOAc. Then, the EtOAc layer was dried in vacuo to give a dark residue (57.30 g). The EtOAc fraction was fractionated by a vacuum liquid silica gel chromatography (90 × 400 mm column, Silica gel 60 H for thin-layer chromatography) using a stepwise gradient of 50–100% hexane/CH_2_Cl_2_ and then 0–50% MeOH/CH_2_Cl_2_ to afford 17 fractions (A–Q). Fraction I was subjected to a Sephadex LH-20 column using an isocratic elution of CH_2_Cl_2_:MeOH (2:1) to yield twelve subfractions (I1–I12), and subfraction I8 was further fractionated by HPLC (Agilent ZORBAX RX-C8, 9.4 × 250 mm, 5 μm column, 3.0 mL/min, elution with 30% to 45% acetonitrile/H_2_O) to yield **1** (2.5 mg) and **2** (4.2 mg). Subfraction I12 was further fractionated by HPLC (Agilent ZORBAX RX-C8, 9.4 × 250 mm, 5 μm column, 3.0 mL/min, elution with 25% to 45% acetonitrile/H_2_O) to yield **3** (1.7 mg) and **11** (2.3 mg). Fraction J was fractionated on a Sephadex LH-20 column using an isocratic elution of CH_2_Cl_2_:MeOH (2:1) to yield fifteen subfractions (J1–J15). Subfraction J6 was further fractionated by HPLC (Agilent ZORBAX RX-C8, 9.4 × 250 mm, 5 μm column, 3.0 mL/min, elution with 10% to 85% acetonitrile/H_2_O) to yield **8** (7.0 mg), **7** (11.0 mg), **10** (10.2 mg), and **9** (6.8 mg). Subfraction J11 was further fractionated by HPLC (Agilent ZORBAX RX-C8, 9.4 × 250 mm, 5 μm column, 3.0 mL/min, elution with 15% to 62% acetonitrile/H_2_O) to yield **12** (7.0 mg). Fraction K was fractionated on a Sephadex LH-20 column using an isocratic elution of CH_2_Cl_2_:MeOH (2:1) to give nine subfractions (K1–K9). Subfraction K4 was further fractionated by HPLC (Agilent ZORBAX RX-C8, 9.4 × 250 mm, 5 μm column, 3.0 mL/min, elution with 19% to 81% acetonitrile/H_2_O) to yield **4** (2.1 mg) and **5** (2.6 mg). Subfraction K9 was further fractionated by HPLC (Agilent ZORBAX RX-C8, 9.4 × 250 mm, 5 μm column, 3.0 mL/min, elution with 15% to 36% acetonitrile/H_2_O) to yield **6** (2.5 mg).

Talamitone A (**1**): Light-yellow oil; ${\left[\alpha \right]}_{D}^{25}$ + 63.5 (*c* 0.00625, MeOH); ^1^H and ^13^C NMR data, Table 1; HRESIMS *m/z* 385.0923 [M + H]^+^ (calcd for C_20_H_17_O_8_, 385.0918).

Talamitone B (**2**): Light-yellow powder; ${\left[\alpha \right]}_{D}^{25}$ + 16.0 (*c* 0.025, MeOH); ^1^H and ^13^C NMR data, Table 1; HRESIMS *m/z* 403.1011 [M + H]^+^ (calcd for C_20_H_19_O_9_, 403.1024).

Demethyltalamitone B (**3**): Light-yellow powder; ${\left[\alpha \right]}_{D}^{25}$ + 12.0 (*c* 0.025, MeOH); ^1^H and ^13^C NMR data, Table 1; HRESIMS *m/z* 389.0871 [M + H]^+^ (calcd for C_19_H_17_O_9_, 389.0867).

Talamiisocoumaringlycoside A (**4**): Light yellow powder; ${\left[\alpha \right]}_{D}^{25}$ + 8.0 (*c* 0.1, MeOH); ^1^H and ^13^C NMR data, Table 2; HRESIMS *m/z* 451.1204 [M + Na]^+^ (calcd for C_19_H_24_O_11_Na, 451.1211).

Talamiisocoumaringlycoside B (**5**): Light yellow powder; ${\left[\alpha \right]}_{D}^{25}$ + 26.0 (*c* 0.1, MeOH); ^1^H and ^13^C NMR data, Table 2; HRESIMS *m/z* 485.0830 [M + Na]^+^ (calcd for C_19_H_23_ClO_11_Na, 485.0821).

Talaminaphtholglycoside (**6**): Light yellow powder; ${\left[\alpha \right]}_{D}^{25}$ + 12.0 (*c* 0.025, MeOH); ^1^H and ^13^C NMR data, Table 2; HRESIMS *m/z* 395.1338 [M + H]^+^ (calcd for C_19_H_23_O_9_, 395.1337).

Mucorisocoumarin B (**7**): ^1^H NMR (500 MHz, DMSO-*d*_6_) *δ*_H_: 11.02 (1H, s), 6.60 (1H, d, *J* = 7.0 Hz), 6.53 (1H, s), 6.51 (1H, d, *J* = 7.0 Hz), 3.96 (1H, m), 3.85 (3H, s), 3.80 (1H, m), 2.64 (1H, dd, *J* = 14.5, 4.0 Hz), 2.46 (1H, dd, *J* = 14.5, 8.5 Hz), 1.58 (1H, m), 1.43 (1H, m), 1.07 (3H, d, *J* = 6.0 Hz); ^13^C NMR (125 MHz, DMSO-*d*_6_) *δ*_C_: 165.7 (C-1), 155.7 (C-3), 105.7 (C-4), 139.7 (C-4a), 101.1 (C-5), 166.5 (C-6), 100.3 (C-7), 162.5 (C-8), 99.4 (C-8a), 41.3 (C-9), 66.2 (C-10), 46.0 (C-11), 64.1 (C-12), 23.7 (C-13), 55.9 (6-OCH_3_); ESIMS *m/z* 293.1 [M − H]^−^.

Diaportinol (**8**): ^1^H NMR (500 MHz, DMSO-*d*_6_) *δ*_H_: 10.97 (1H, s), 6.59 (1H, d, *J* = 2.5 Hz), 6.54 (1H, s), 6.51 (1H, d, *J* = 2.5 Hz), 4.89 (1H, d, *J* = 5.0 Hz), 4.73 (1H, t, *J* = 5.5 Hz), 3.85 (3H, s), 3.81 (1H, m), 3.40 (1H, m), 3.30 (1H, m), 2.74 (1H, dd, *J* = 14.5, 3.5 Hz), 2.39 (1H, dd, *J* = 14.5, 9.0 Hz); ^13^C NMR (125 MHz, DMSO-*d*_6_) *δ*_C_: 165.6 (C-1), 155.9 (C-3), 105.5 (C-4), 139.7 (C-4a), 101.1 (C-5), 166.5 (C-6), 100.3 (C-7), 162.5 (C-8), 99.4 (C-8a), 37.8 (C-9), 69.0 (C-10), 65.4 (C-11), 55.9 (6-OCH_3_); ESIMS *m/z* 267.1 [M + H]^+^.

Peniisocoumarin E (**9**): ^1^H NMR (500 MHz, DMSO-*d*_6_) *δ*_H_: 10.93 (1H, s), 6.65 (1H, s), 6.63 (1H, d, *J* = 2.5 Hz), 6.55 (1H, d, *J* = 2.5 Hz), 4.30 (1H, m), 3.85 (3H, s), 3.66 (2H, m), 3.17 (1H, dd, *J* = 15.0, 10.0 Hz), 2.78 (1H, dd, *J* = 15.0, 4.0 Hz); ^13^C NMR (125 MHz, DMSO-*d*_6_) *δ*_C_: 165.1 (C-1), 153.7 (C-3), 106.2 (C-4), 139.2 (C-4a), 101.5 (C-5), 166.6 (C-6), 100.8 (C-7), 162.6 (C-8), 99.4 (C-8a), 37.8 (C-9), 60.2 (C-10), 65.0 (C-11), 56.0 (6-OCH_3_); ESIMS *m/z* 285.1 [M + H]^+^.

Dichlorodiaportin (**10**): ^1^H NMR (500 MHz, DMSO-*d*_6_) *δ*_H_: 10.97 (1H, s), 6.62 (1H, s), 6.61 (1H, d, *J* = 2.5 Hz), 6.54 (1H, d, *J* = 2.5 Hz), 6.31 (1H, d, *J* = 3.0 Hz), 6.13 (1H, d, *J* = 6.0 Hz), 4.18 (1H, m), 3.85 (3H, s), 2.88 (1H, dd, *J* = 14.5, 3.0 Hz), 2.64 (1H, dd, *J* = 14.5, 9.5 Hz); ^13^C NMR (125 MHz, DMSO-*d*_6_) *δ*_C_: 165.3 (C-1), 153.7 (C-3), 106.3 (C-4), 139.4 (C-4a), 101.4 (C-5), 166.5 (C-6), 100.5 (C-7), 162.5 (C-8), 99.5 (C-8a), 36.4 (C-9), 72.2 (C-10), 77.0 (C-11), 56.0 (6-OCH_3_); ESIMS *m/z* 319.1 [M + H]^+^.

Orsellinic acid (**11**): ^1^H NMR (500 MHz, CD_3_OD) *δ*_H_: 6.19 (1H, d, *J* = 2.5 Hz), 6.13 (1H, d, *J* = 2.5 Hz), 2.49 (3H, s); ^13^C NMR (125 MHz, CD_3_OD) *δ*_C_: 105.1 (C-1), 163.6 (C-2), 101.7 (C-3), 166.9 (C-4), 112.2 (C-5), 145.2 (C-6), 175.2 (C-7), 24.3 (8-CH_3_); ESIMS *m/z* 169.1 [M + H]^+^.

4-(Hydroxymethyl)-5-hydroxy-2*H*-pyran-2-one (**12**): ^1^H NMR (500 MHz, DMSO-*d*_6_) *δ*_H_: 8.03 (1H, s), 6.33 (1H, s), 4.28 (2H, s); ^13^C NMR (125 MHz, DMSO-*d*_6_) *δ*_C_: 174.0 (C-2), 109.8 (C-3), 145.7 (C-4), 168.1 (C-5), 139.3 (C-6), 59.5 (C-7).; ESIMS *m/z* 143.0 [M + H]^+^.

### 3.3. Biological Activity

Compounds **1**–**12** were evaluated against *S*. *aureus*, *E*. *coli*, and *C*. *albicans* for their antimicrobial activities in 96-well plates according to the Antimicrobial Susceptibility Testing Standards outlined by the Clinical and Laboratory Standards Institute document M07-A7 (CLSI) [29] and our previous report [30,31]. The MIC was defined as the minimum concentration of the compound that prevented visible growth of the microbes. The synergistic antimicrobial activities were determined by combing one-fourth of the minimum inhibitory concentration of the positive control (MIC of methicillin was 0.5 μg/mL) for *S. aureus* with two-fold diluted tested compounds. The synergistic antimicrobial activities were calculated based on a previous report [32]. Briefly, final concentrations were preprepared from 1.5625 to 100 μg/mL of isolated compounds. Methicillin was added by a column, while the tested compounds were diluted 2-fold by row in a 96-well microtiter plate. The fractional inhibitory concentration index (FICI) was calculated as the sum of the MIC of each drug when used in combination, divided by the MIC of the drug used alone. Synergistic antibacterial activity was defined by FICI ≤ 0.5.

## 4. Conclusions

In summary, 12 compounds, including 6 new compounds, talamitones A and B (**1** and **2**), demethyltalamitone B (**3**), talamiisocoumaringlycosides s A and B (**4** and **5**), and talaminaphtholglycoside (**6**), together with 6 previous reported compounds, mucorisocoumarin B (**7**), diaportinol (**8**), peniisocoumarin E (**9**), dichlorodiaportin (**10**), orsellinic acid (**11**), and 4-(hydroxymethyl)-5-hydroxy-2*H*-pyran-2-one (**12**), were isolated from the marine sediment-derived fungus *T. minnesotensis* BTBU20220184. The structures of the new compounds were characterized by HREIMS and NMR data analysis. All of the compounds were screened for activity against *S. aureus*, *E. coli*, and *C. albicans*. Compound **8** exhibited weak antibacterial activity against *S. aureus* at a concentration of 200 μg/mL. Compounds **5**, **6,** and **9** showed synergistic antibacterial activity against *S. aureus* at concentrations of 25, 50, and 12.5 μg/mL with 0.125 μg/mL of methicillin.

## Figures and Tables

**Table 1:** ^1^H (500 MHz) and ^13^C NMR (125 MHz) data of **1**–**3** (CD_3_OD).

Pos	1		2		3	
	*δ*_C_, Type	*δ*_H,_ (*J* in Hz)	*δ*_C_, Type	*δ*_H,_ (*J* in Hz)	*δ*_C_, Type	*δ*_H,_ (*J* in Hz)
1	198.5, C		201.0, C		201.2, C	
2	136.7, C		38.8, CH_2_	3.06, t (7.0)	38.8, CH_2_	3.06, t (7.0)
3	154.0, C		202.8, C		202.9, C	
3a	134.8, C		136.8, C		136.5, C	
4	152.6, C		155.2, C		155.2, C	
5	126.8, CH	7.50, d (1.5)	121.7, C	7.68, d (1.0)	121.8, CH	7.68, d (1.5)
6	134.4, C		132.8, C		133.7, C	
7	115.4, CH	7.51, d (1.5)	122.0, C	8.07, d (1.0)	122.0, CH	8.08, d (1.5)
7a	133.1, C		137.3, C		137.3, C	
8	20.7, CH_2_	2.44, m	20.4, CH_2_	1.90, quint (7.0)	20.5, CH_2_	1.90, quint (7.0)
9	32.9, CH_2_	2.46, m	33.7, CH_2_	2.35, t (7.0)	33.9, CH_2_	2.35, t (7.0)
10	175.3, C		175.5, C		177.2, C	
11	168.9, C		168.6, C		169.3, C	
1′	110.7, C		112.2, C		112.2, C	
2′	156.7, C		163.3, C		163.3, C	
3′	107.6, CH	6.39, d (8.0)	108.1, CH	6.28, d (8.5)	108.1, CH	6.28, d (8.0)
4′	130.9, CH	7.04, t (8.0)	137.4, CH	7.21, t (8.5)	137.4, CH	7.21, t (8.0)
5′	107.6, CH	6.39, d (8.0)	108.1, CH	6.28, d (8.5)	108.1, CH	6.28, d (8.0)
6′	156.7, C		163.3, C		163.3, C	
10-OCH_3_	52.1, CH_3_	3.60, s	52.1, CH_3_	3.64, s		

**Table 2:** ^1^H (500 MHz) and ^13^C NMR (125 MHz) data of **4**–**6** (CD_3_OD).

Pos	4		5		6	
	*δ*_C_, Type	*δ*_H_, (*J* in Hz)	*δ*_C_, Type	*δ*_H_, (*J* in Hz)	*δ*_C_, Type	*δ*_H_, (*J* in Hz)
1	167.9, C		167.5, C		110.0, CH	6.89, s
2	-		-		158.4, C	
3	157.9, C		154.0, C		119.5, C	
4	101.2, CH	6.94, s	103.6, CH	7.04, s	131.3, CH	9.17, s
4a	133.5, C		133.0, C		118.6, C	
5	132.9, C		133.2, C		154.0, C	
6	161.1, C		161.1, C		120.7, C	
7	99.7, CH	6.63, s	100.1, CH	6.66, s	160.5, C	
8	161.8, C		161.9, C		104.4, CH	6.76, s
8a	99.3, C		99.3, C		140.4, C	
9a	30.7, CH_2_	2.63, m	39.4, CH_2_	3.15, dd (15.0, 4.0)	207.0, C	
9b				2.88, dd (15.0, 9.5)		
10	30.8, CH_2_	1.90, tt (7.0)	60.4, CH	4.32, m	27.2, CH_3_	2.76, s
11	61.7, CH_2_	3.63, m	66.8, CH_2_	3.78, m	10.9, CH_3_	2.34, s
1′	105.9, CH	4.70, d (7.5)	106.0, CH	4.70, d (7.5)	107.1, CH	4.78, d (7.5)
2′	75.6, CH	3.50, m	75.6, CH	3.51, dd (9.0, 7.5)	75.8, CH	3.69, m
3′	77.8, CH	3.42, m	77.8, CH	3.44, m	78.2, CH	3.47, m
4′	71.2, CH	3.42, m	71.2, CH	3.44, m	71.4, CH	3.47, m
5′	78.2, CH	3.17, m	78.1, CH	3.17, m	77.9, CH	3.13, m
6′a	62.5, CH_2_	3.76, dd (11.5, 2.5)	62.4, CH_2_	3.76, dd (11.5, 2.5)	62.6, CH_2_	3.76, dd (11.5, 2.5)
6′b		3.66, dd (11.5, 5.0)		3.67, dd (11.5, 4.5)		3.67, m
6-OCH_3_	56.8, CH_3_	3.94, s	56.9, CH_3_	3.94, s		

## Data Availability

The data presented in this study are available on request from the corresponding author.

## Supplementary Materials

The following supporting information can be downloaded at https://www.mdpi.com/article/10.3390/md22060237/s1, Figures S1–S6: HRESIMS, ^1^H, ^13^C, HSQC, ^1^H–^1^H COSY, and HMBC spectra for compound **1;** Figures S7–S12: HRESIMS, ^1^H, ^13^C, HSQC, ^1^H–^1^H COSY, and HMBC spectra for compound **2;** Figures S13–S18: HRESIMS, ^1^H, ^13^C, HSQC, ^1^H–^1^H COSY, and HMBC spectra for compound **3;** Figures S19–S24: HRESIMS, ^1^H, ^13^C, HSQC, ^1^H–^1^H COSY, and HMBC spectra for compound **4**; Figures S25–S30: HRESIMS, ^1^H, ^13^C, HSQC, ^1^H–^1^H COSY, and HMBC spectra for compound **5**; Figures S31–S37: HRESIMS, ^1^H, ^13^C, HSQC, ^1^H–^1^H COSY, HMBC, and ROESY spectra for compound **6**; Figure S38: Phylogenetic tree of BTBU20220184..
