# Supplementary material for: New Secondary Metabolites from Marine-Derived Fungus Talaromyces minnesotensis BTBU20220184
Source: Mar Drugs. 2024 May 23;22(6):237. doi: 10.3390/md22060237 (PMC11204780; doi:10.3390/md22060237)
Supplement: Supplementary file 1 [file marinedrugs-22-00237-s001.zip › marinedrugs-3013240-supplementary.pdf]

SUPPLEMENTARY MATERIAL

New Secondary Metabolites from Marine-Derived Fungus *Talaromyces minnesotensis* BTBU20220184

Weiliang Wang [1], Jingjing Wang [1], Fuhang Song [2], Renming Jia [3], Long Wang [4], Xiuli Xu [1,*] and Na Yang [5,6,*]

[1] Key Laboratory of Marine Mineral Resources and Polar Geology, Ministry of Education, School of Ocean Sciences, China University of Geosciences, Beijing 100083, China; wxw1134@126.com (W.W.); jingjingwang202404@163.com (J.W.)

[2] Key Laboratory of Geriatric Nutrition and Health, Ministry of Education of China, School of Light Industry Science and Engineering, Beijing Technology and Business University, Beijing 100048, China;
songfuhang@btbu.edu.cn

[3] Key Laboratory of Tropical Marine Ecosystem and Bioresource, Guangxi Key Laboratory of Beibu Gulf Marine Resources, Environment and Sustainable Development, Fourth Institute of Oceanography,
Ministry of Natural Resources, Beihai, 536000, China; jiarenming@4io.org.cn

[4] State Key Laboratory of Mycology, Institute of Microbiology, Chinese Academy of Sciences, Beijing 100101, China; wl_dgk@sina.com

[5] CAS Key Laboratory of Experimental Marine Biology, Center for Ocean Mega-Science, Institute of Oceanology, Chinese Academy of Sciences, Qingdao 266071, China

[6] Laboratory for Marine Biology and Biotechnology, Qingdao National Laboratory for Marine Science
and Technology, Qingdao 266237, China

* Correspondence: xuxl@cugb.edu.cn (X.X.); yangna@qdio.ac.cn (N.Y.)

Table of Contents

Figure S1. HRESIMS spectrum for **1** 3
Figure S2. ^{1}H NMR spectrum (500 MHz, CD_3OD) of **1** 3
Figure S3. ^{13}C NMR spectrum (125 MHz, CD_3OD) of **1** 4
Figure S4. HSQC spectrum (500 MHz, CD_3OD) of **1** 4
Figure S5. ^{1}H -^{1}H COSY spectrum (500 MHz, CD_3OD) of **1** 5
Figure S6. HMBC spectrum (500 MHz, CD_3OD) of **1** 5
Figure S7. HRESIMS spectrum for **2** 6
Figure S8. ^{1}H NMR spectrum (500 MHz, CD_3OD) of **2** 6
Figure S9. ^{13}C NMR spectrum (125 MHz, CD_3OD) of **2** 7
Figure S10. HSQC spectrum (500 MHz, CD_3OD) of **2** 7
Figure S11. ^{1}H -^{1}H COSY spectrum (500MHz, CD_3OD) of **2** 8
Figure S12. HMBC spectrum (500MHz, CD_3OD) of **2** 8
Figure S13. HRESIMS spectrum for **3** 9
Figure S14. ^{1}H NMR spectrum (500 MHz, CD_3OD) of **3** 9
Figure S15. ^{13}C NMR spectrum (125 MHz, CD_3OD) of **3** 10
Figure S16. HSQC spectrum (500 MHz, CD_3OD) of **3** 10
Figure S17. ^{1}H -^{1}H COSY spectrum (500MHz, CD_3OD) of **2** 11
Figure S18. HMBC spectrum (500 MHz, CD_3OD) of **3** 11
Figure S19. HRESIMS spectrum for **4** 12
Figure S20. ^{1}H NMR spectrum (500 MHz, CD_3OD) of **4** 12
Figure S21. ^{13}C NMR spectrum (125 MHz, CD_3OD) of **4** 13
Figure S22. ^{1}H -^{1}H COSY spectrum (500MHz, CD_3OD) of **2** 13
Figure S23. HSQC spectrum (500 MHz, CD_3OD) of **4** 14
Figure S24. HMBC spectrum (500 MHz, CD_3OD) of **4** 14
Figure S25. HRESIMS spectrum for **5** 15
Figure S26. ^{1}H NMR spectrum (500 MHz, CD_3OD) of **5** 15
Figure S27. ^{13}C NMR spectrum (125 MHz, CD_3OD) of **5** 16
Figure S28. HSQC spectrum (500 MHz, CD_3OD) of **5** 16
Figure S29. ^{1}H -^{1}H COSY spectrum (500 MHz, CD_3OD) of **5** 17
Figure S30. HMBC spectrum (500 MHz, CD3OD) of **5** 17
Figure S31. HRESIMS spectrum for **6** 18
Figure S32. ^{1}H NMR spectrum (500 MHz, CD_3OD) of **6** 18
Figure S33. ^{13}C NMR spectrum (125 MHz, CD_3OD) of **6** 19
Figure S34. HSQC spectrum (500 MHz, CD_3OD) of **6** 19
Figure S35. ^{1}H -^{1}H COSY spectrum (500 MHz, CD_3OD) of **6** 20
Figure S36. HMBC spectrum (500 MHz, CD3OD) of **6** 20
Figure S37. ROESY spectrum (500 MHz, CD3OD) of **6** 21
Figure S38. Phylogenetic tree of BTBU20220184 21

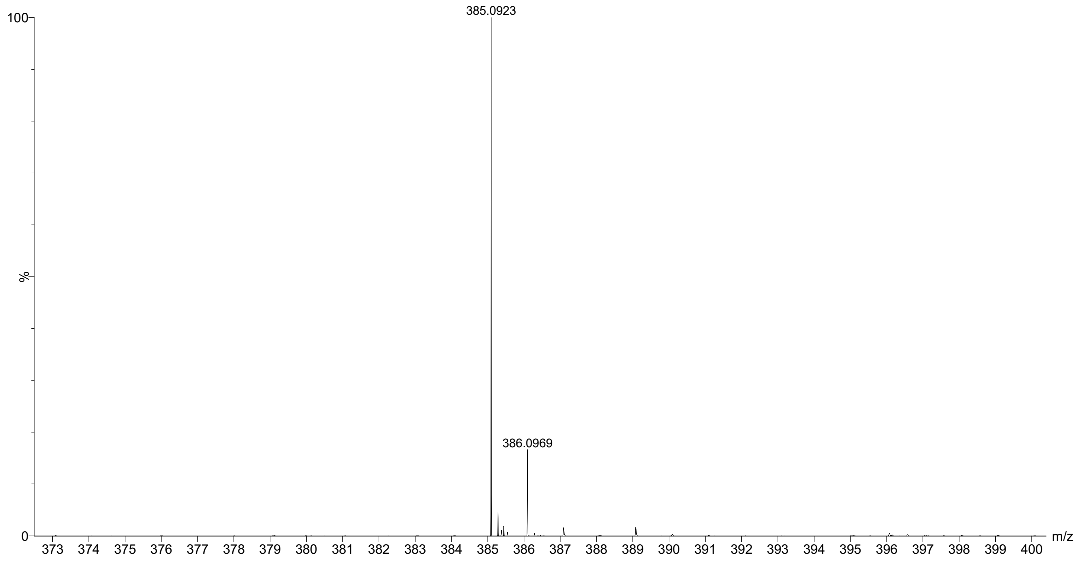

Figure S1. HRESIMS spectrum for compound **1**

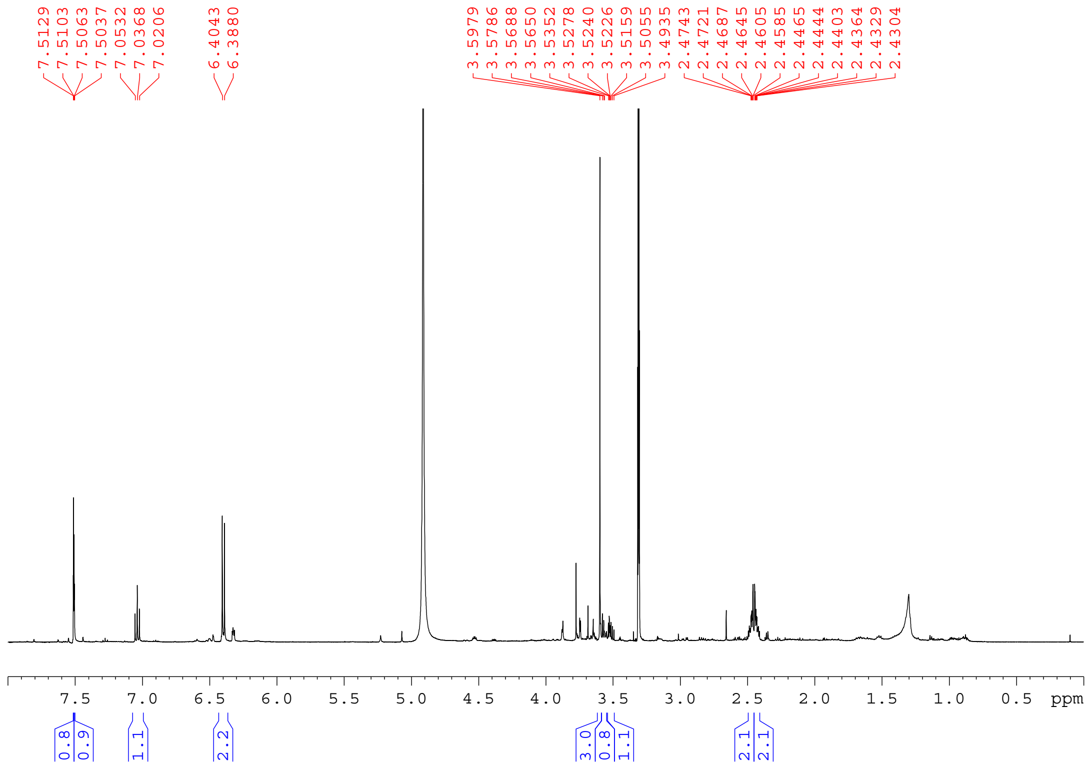

Figure S2. ^{1}H NMR spectrum (500 MHz, CD_3OD) of **1**

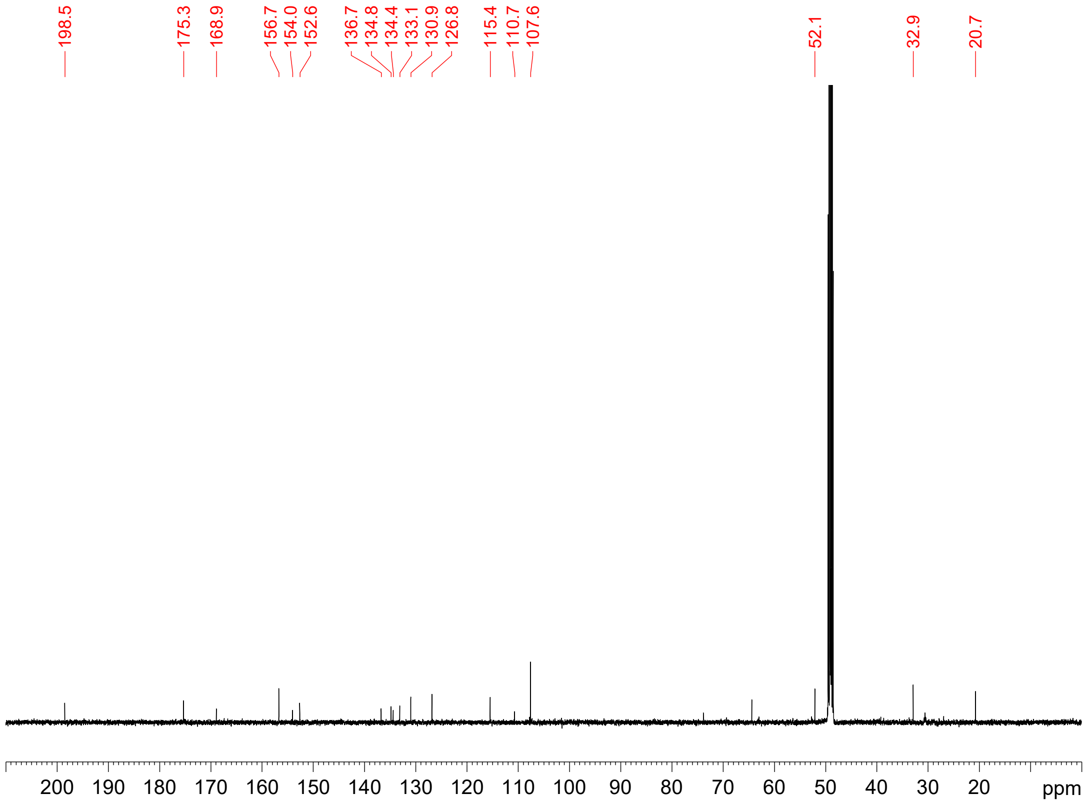

Figure S3. ^{13}C NMR spectrum (125 MHz, CD_3OD) of **1**

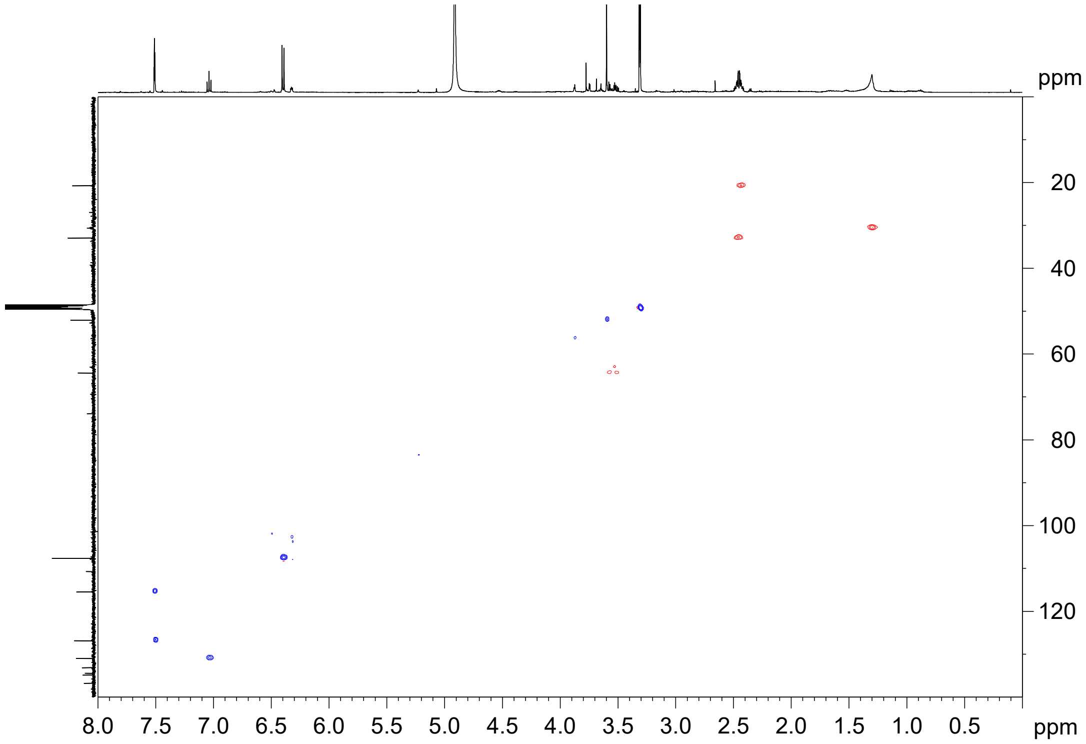

Figure S4. HSQC spectrum (500 MHz, CD_3OD) of **1**

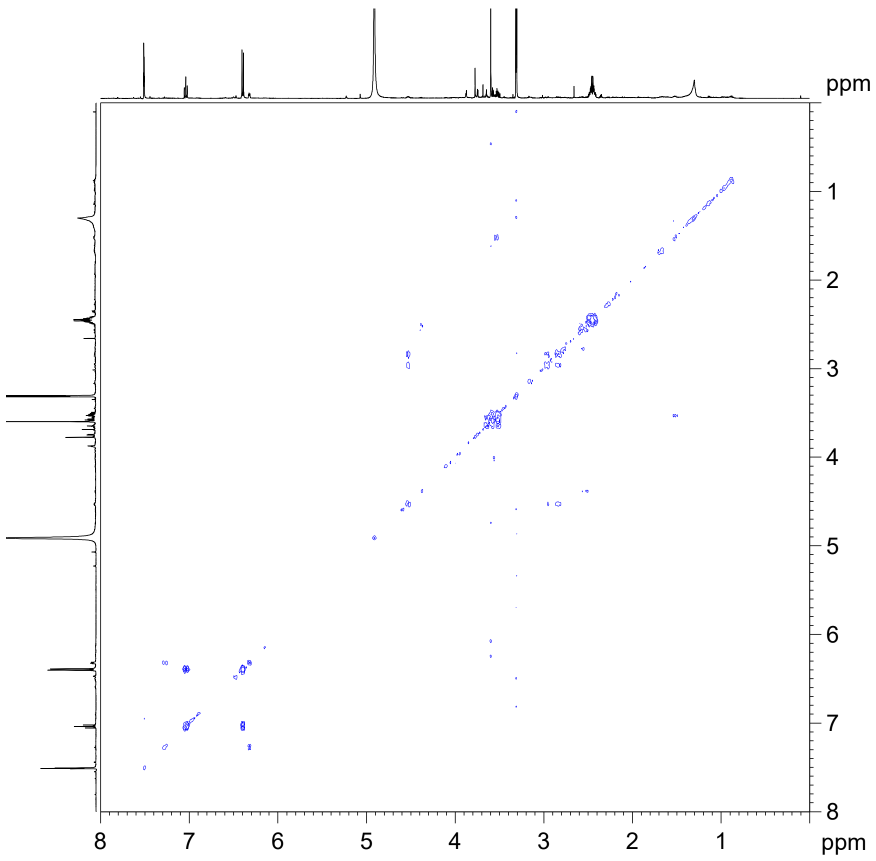

Figure S5. ^{1}H -^{1}H COSY spectrum (500 MHz, CD_3OD) of **1**

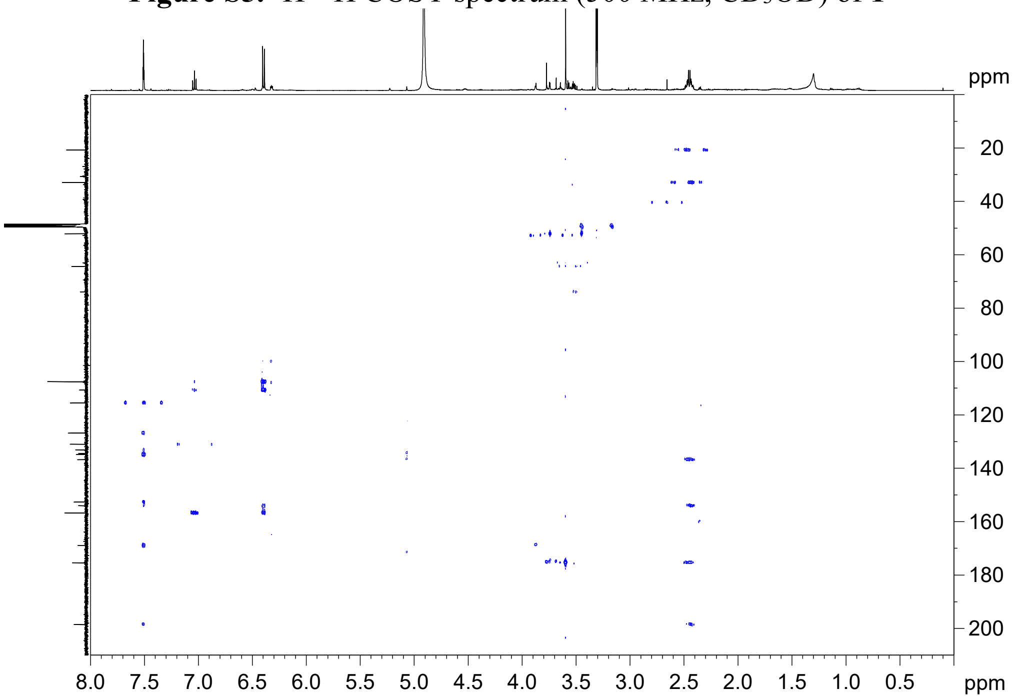

Figure S6. HMBC spectrum (500 MHz, CD_3OD) of **1**

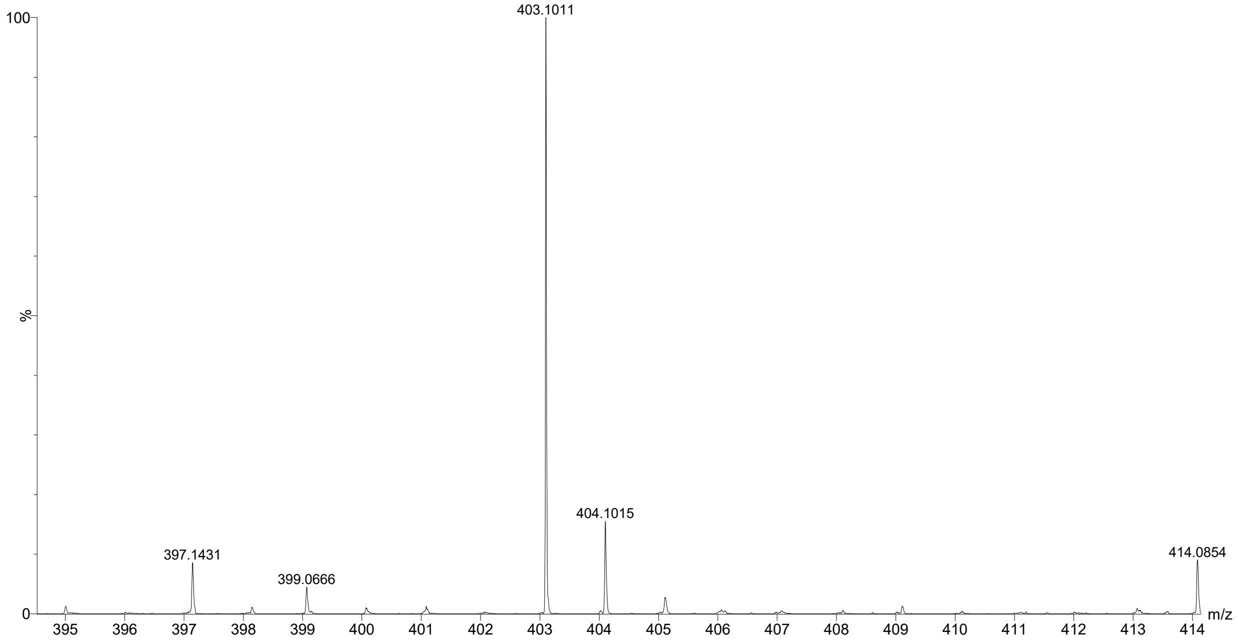

Figure S7. HRESIMS spectrum for compound **2**

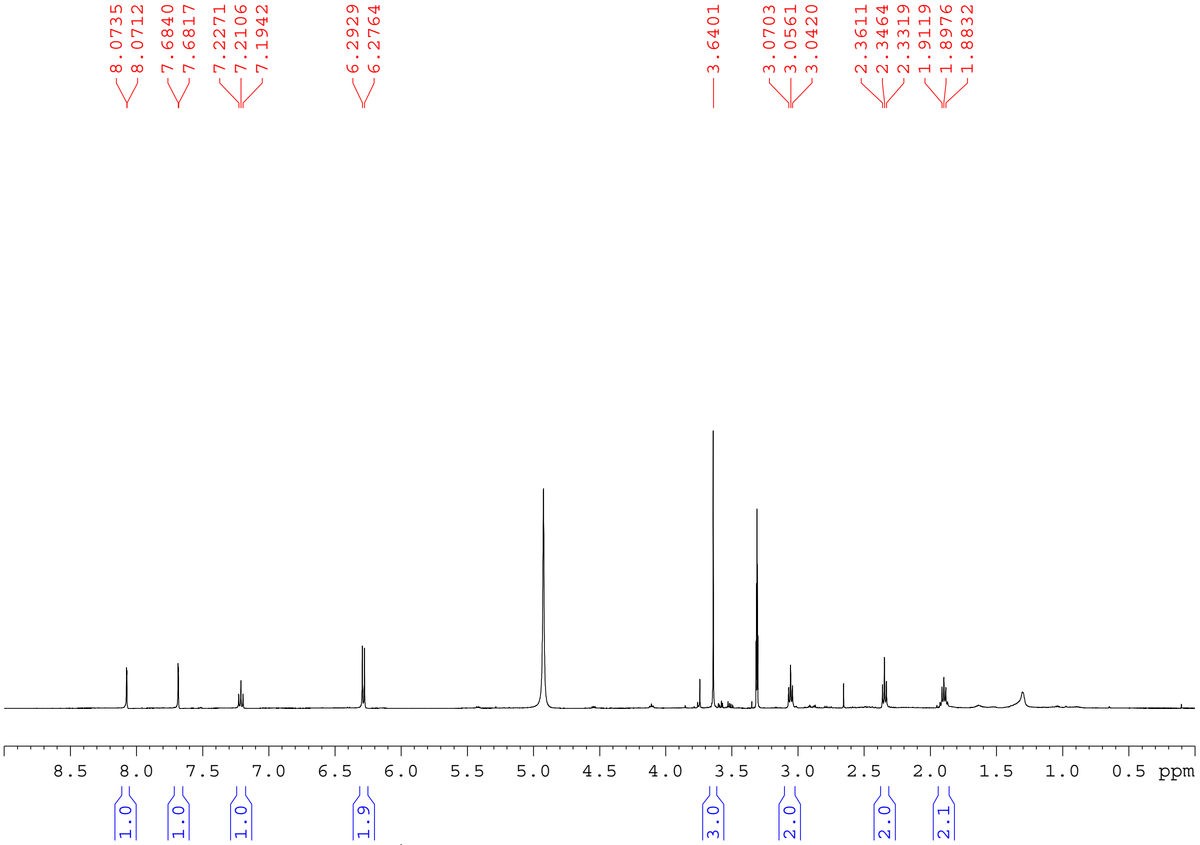

Figure S8. ^{1}H NMR spectrum (500 MHz, CD_3OD) of **2**

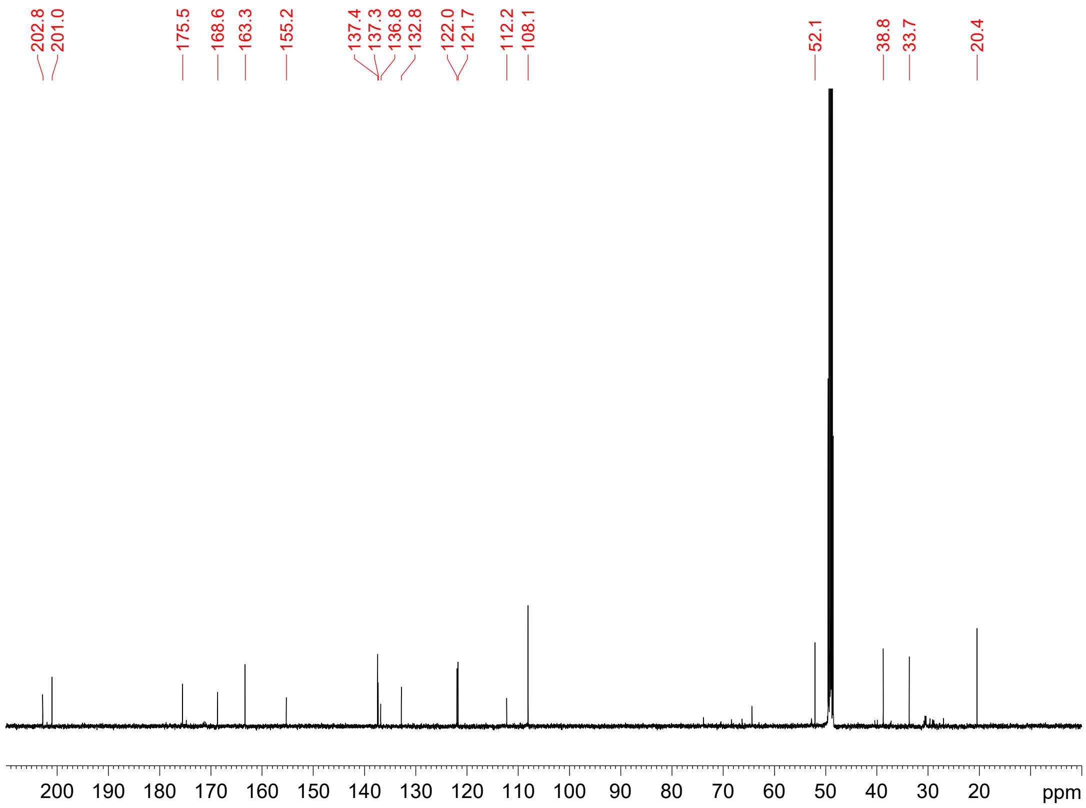

Figure S9. ^{13}C NMR spectrum (125 MHz, CD_3OD) of **2**

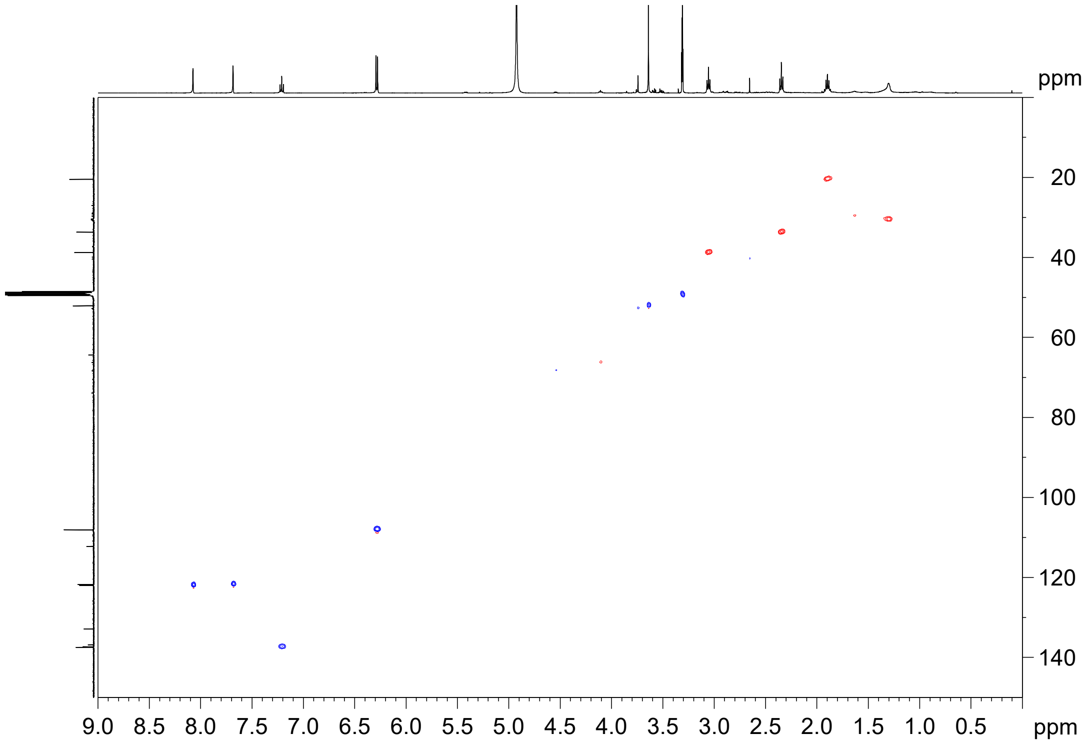

Figure S10. HSQC spectrum (500 MHz, CD_3OD) of **2**

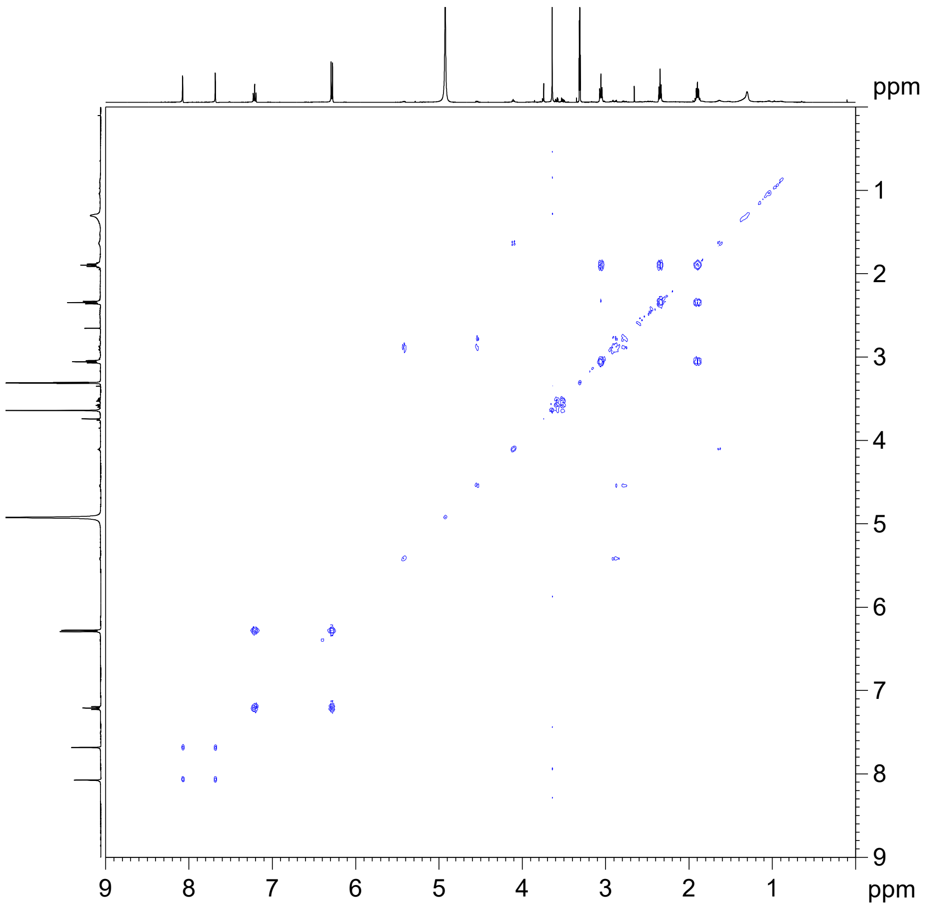

Figure S11. ^{1}H -^{1}H COSY spectrum (500 MHz, CD_3OD) of **2**

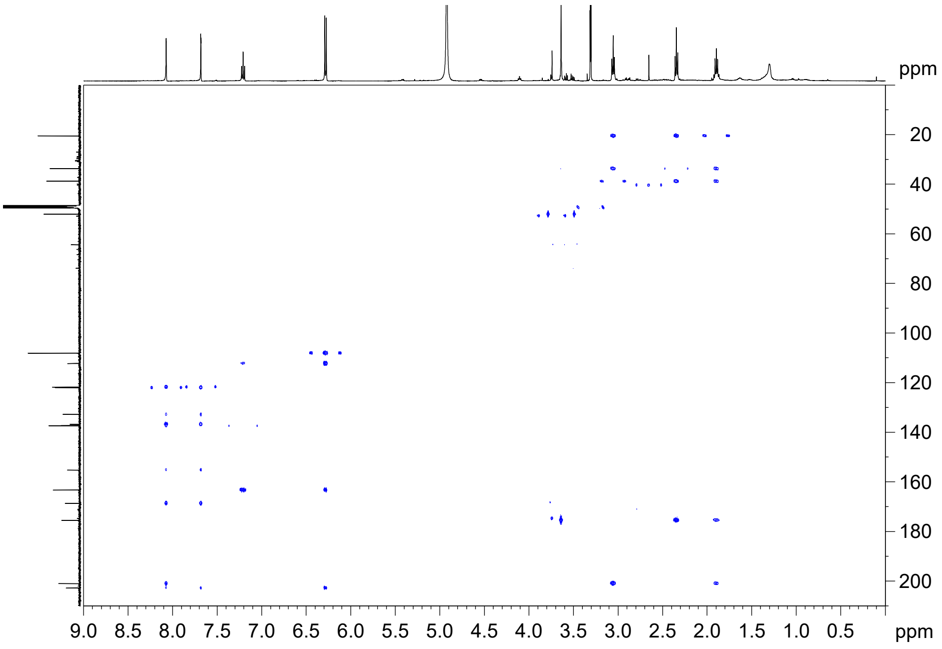

Figure S12. HMBC spectrum (500 MHz, CD_3OD) of **2**

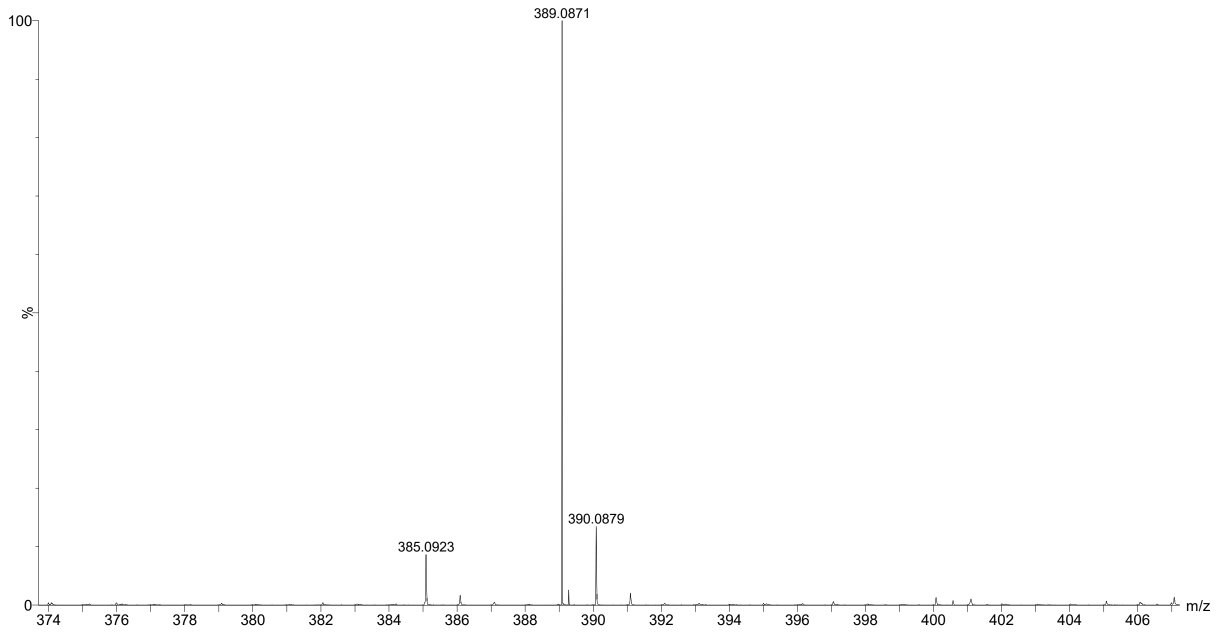

Figure S13. HRESIMS spectrum for compound **3**

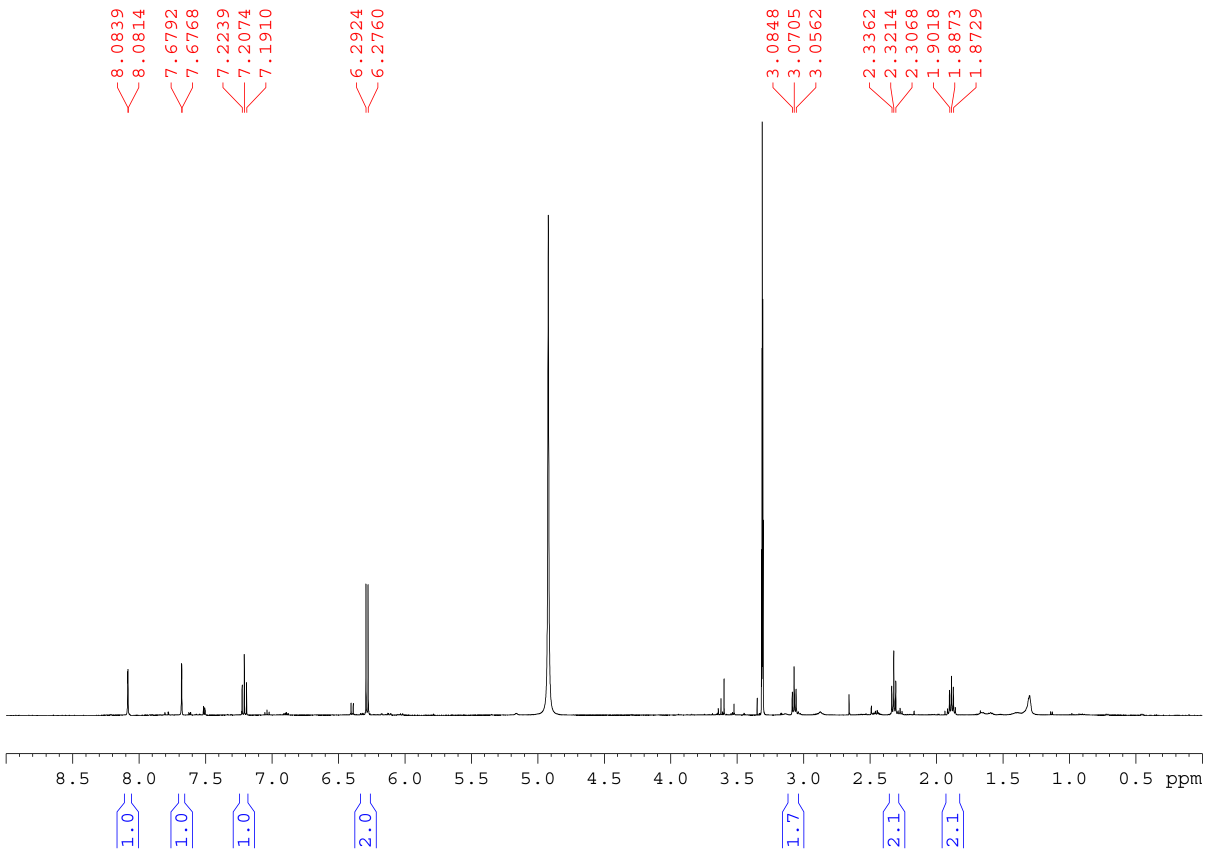

Figure S14. ^{1}H NMR spectrum (500 MHz, CD_3OD) of **3**

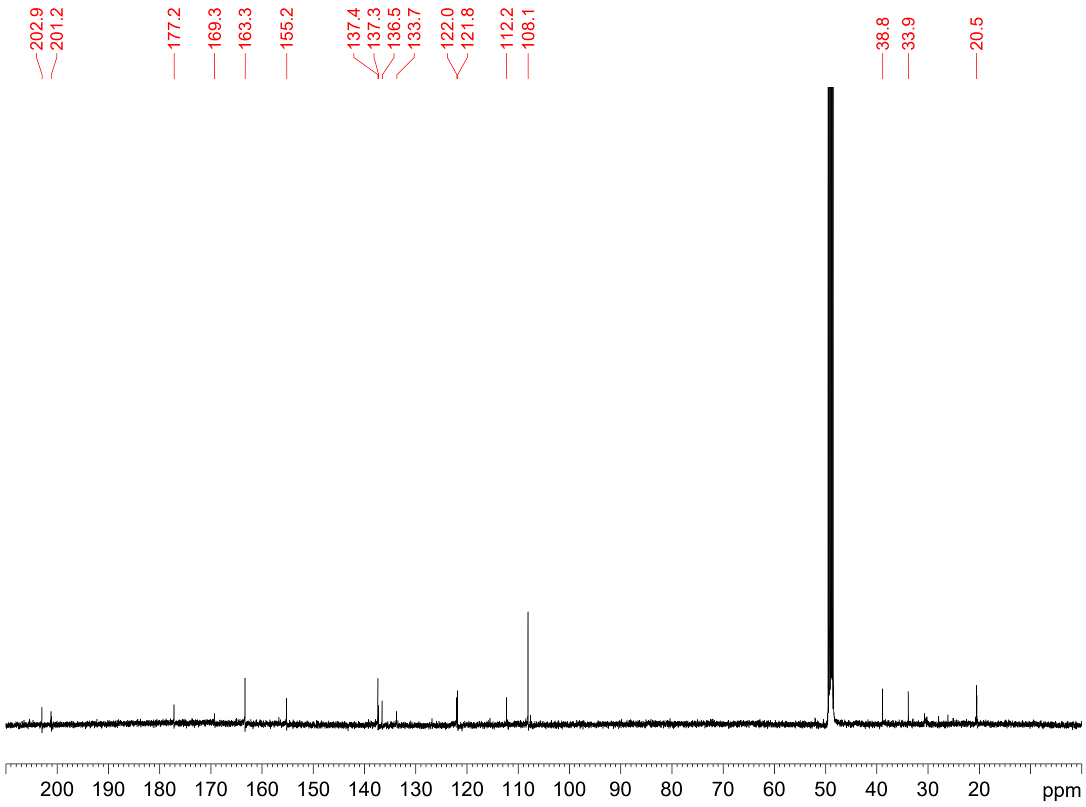

Figure S15. ^{13}C NMR spectrum (125 MHz, CD_3OD) of **3**

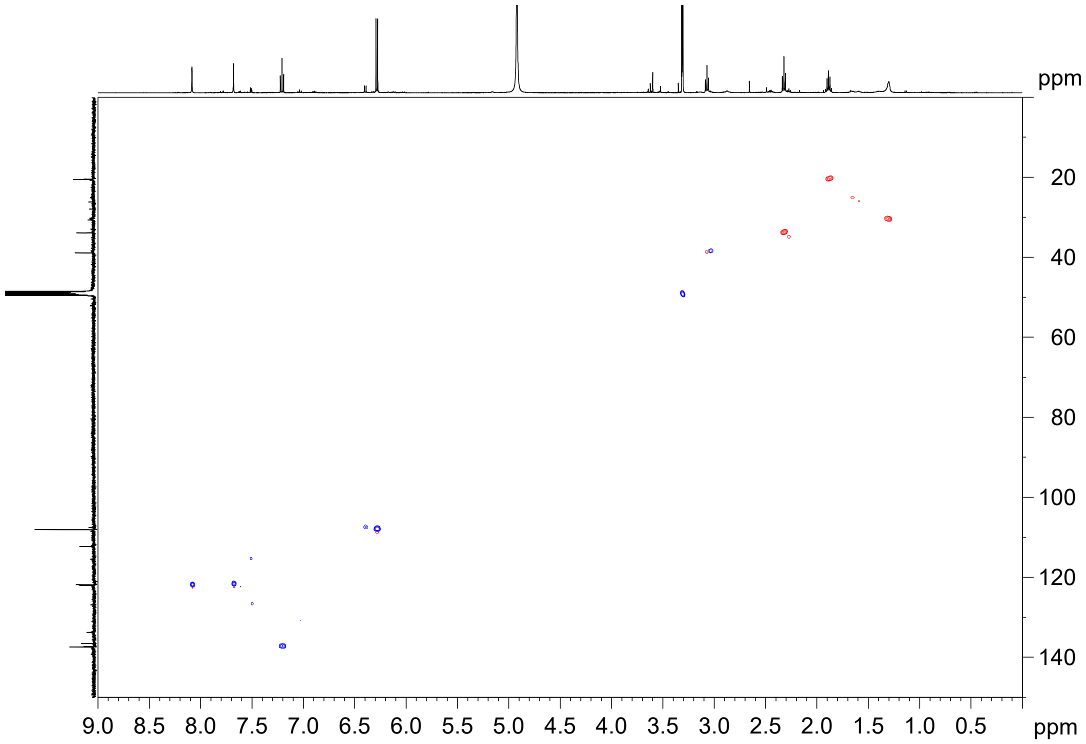

Figure S16. HSQC spectrum (500 MHz, CD_3OD) of **3**

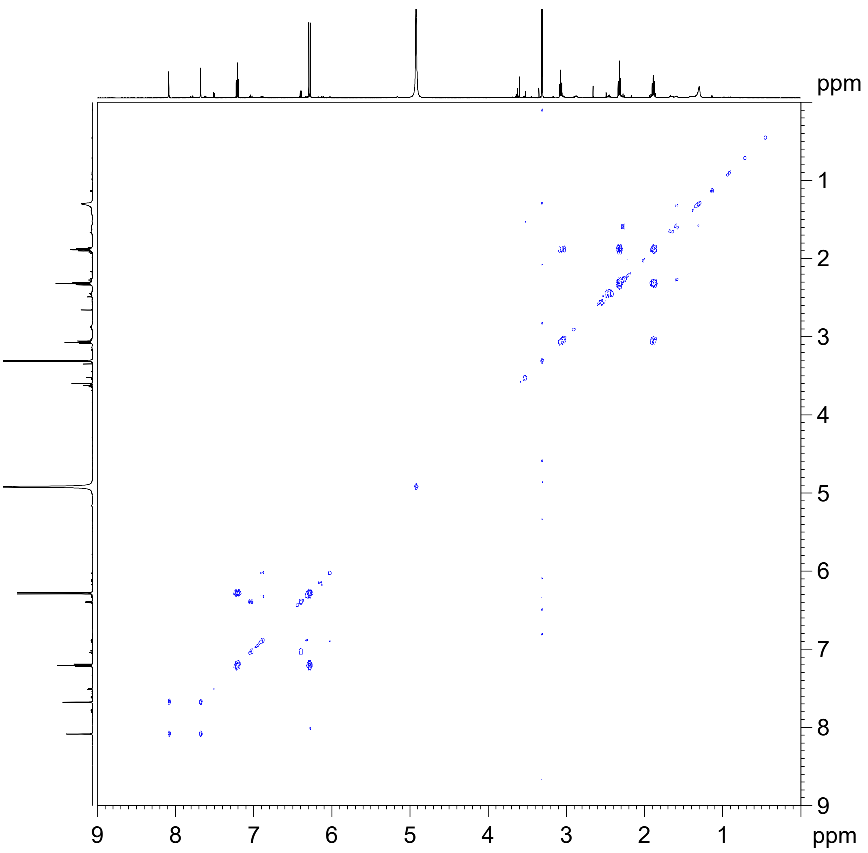

Figure S17. ^1H -^1H COSY spectrum (500 MHz, CD_3OD) of **3**

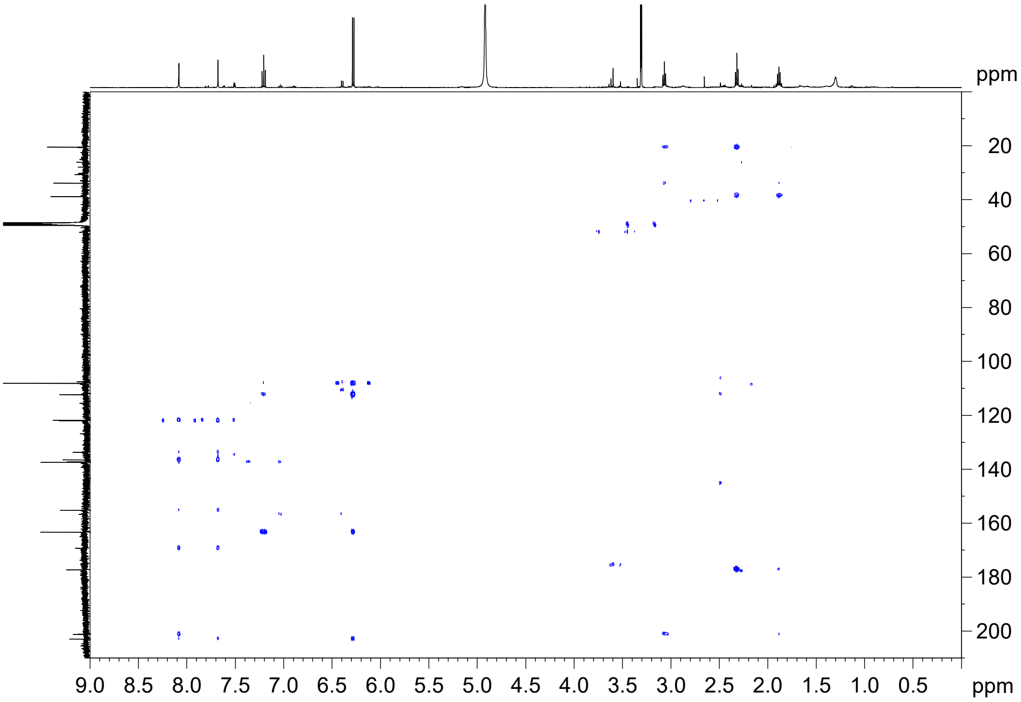

Figure S18. HMBC spectrum (500 MHz, CD_3OD) of **3**

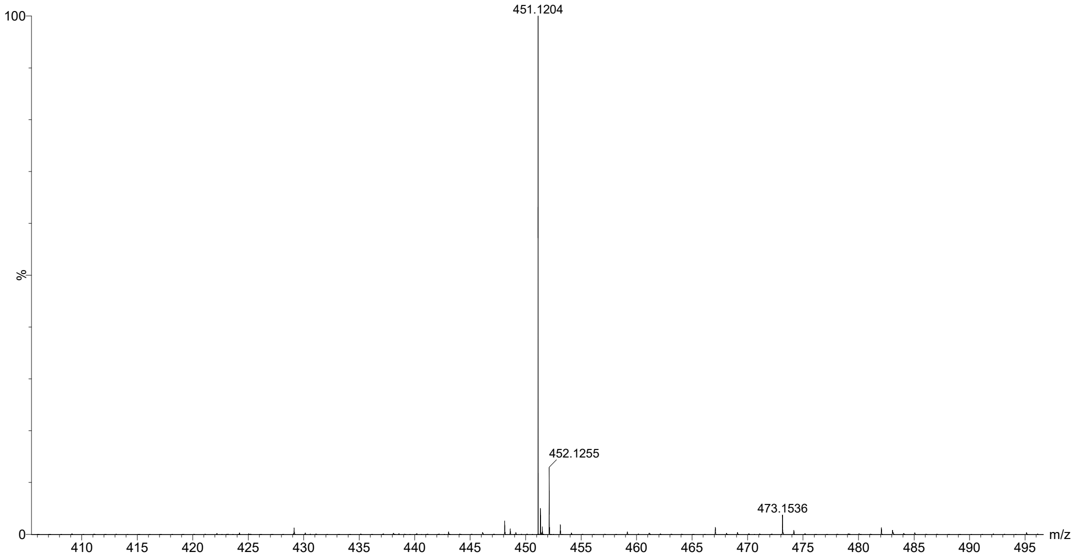

Figure S19. HRESIMS spectrum for compound **4**

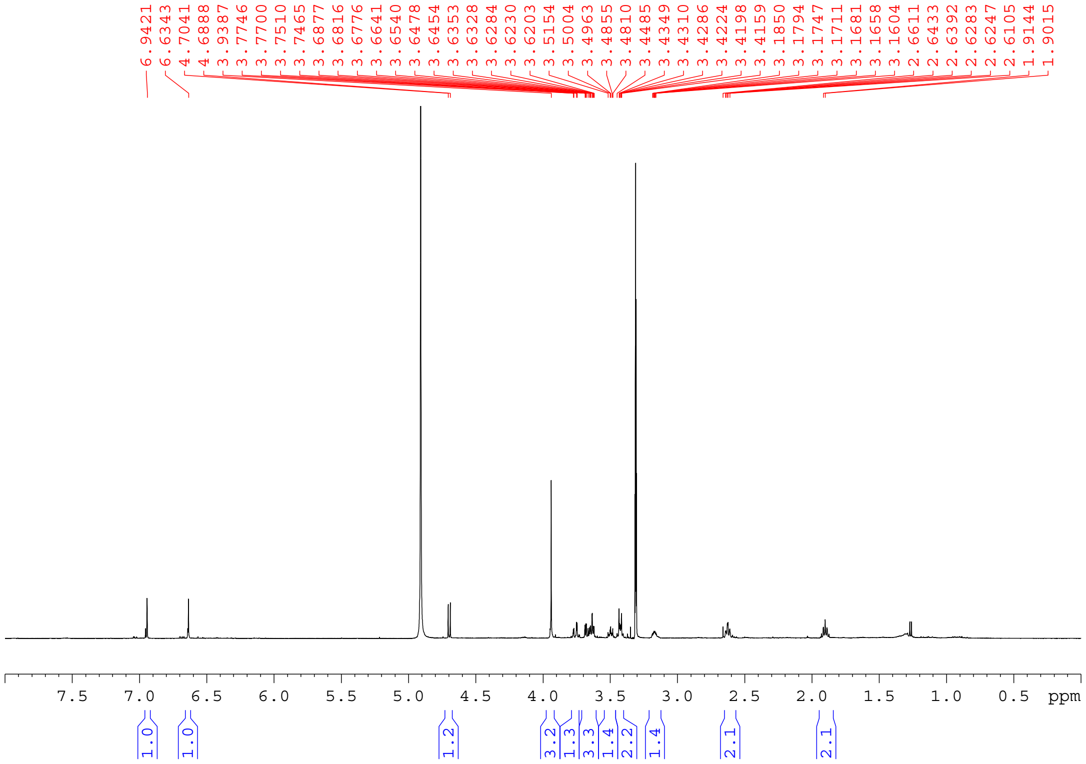

Figure S20. ^{1}H NMR spectrum (500 MHz, CD_3OD) of **4**

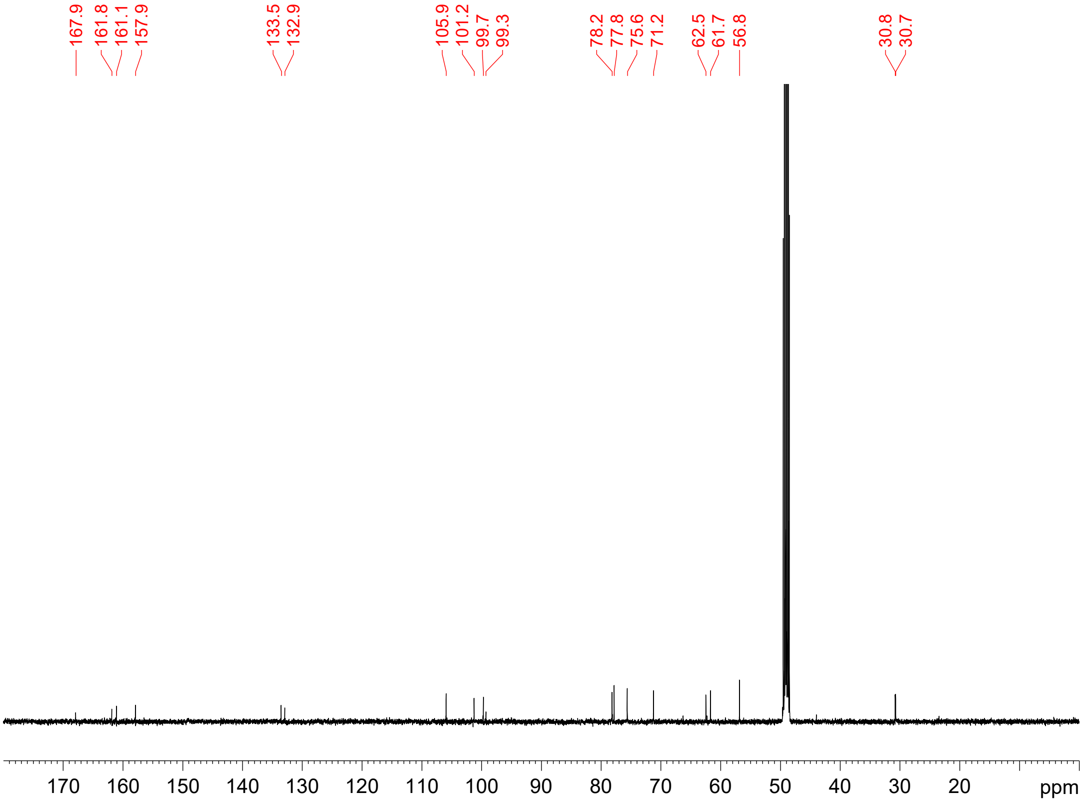

Figure S21. ^{13}C NMR spectrum (125 MHz, CD_3OD) of **4**

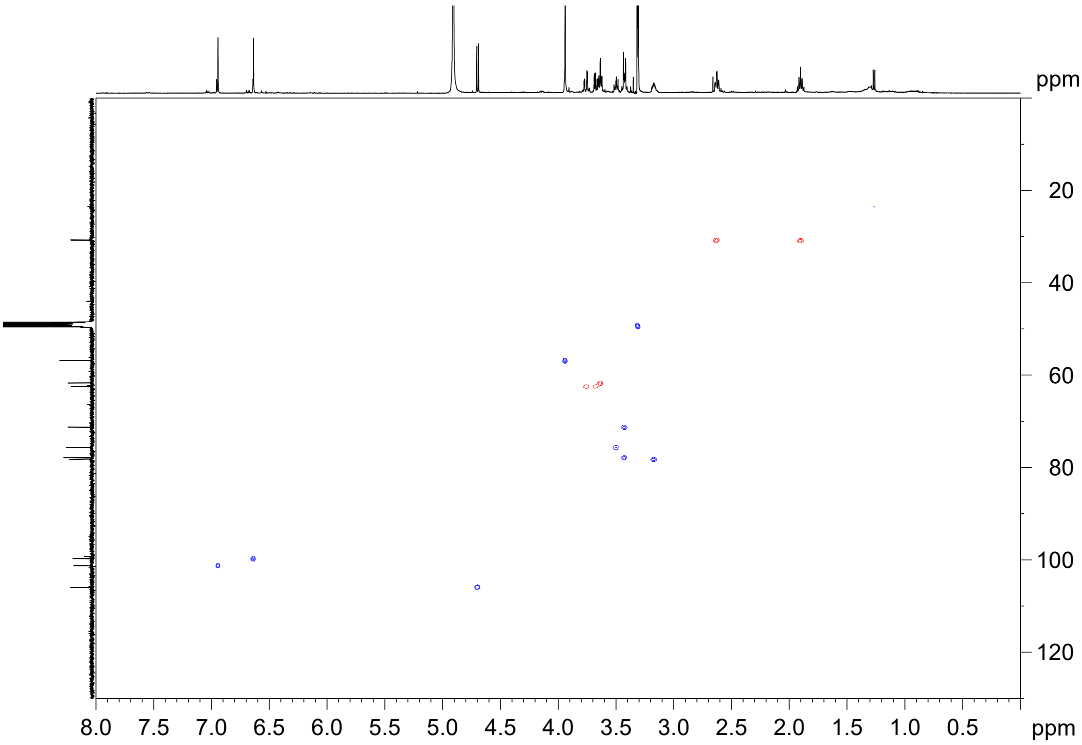

Figure S22. HSQC spectrum (500 MHz, CD_3OD) of **4**

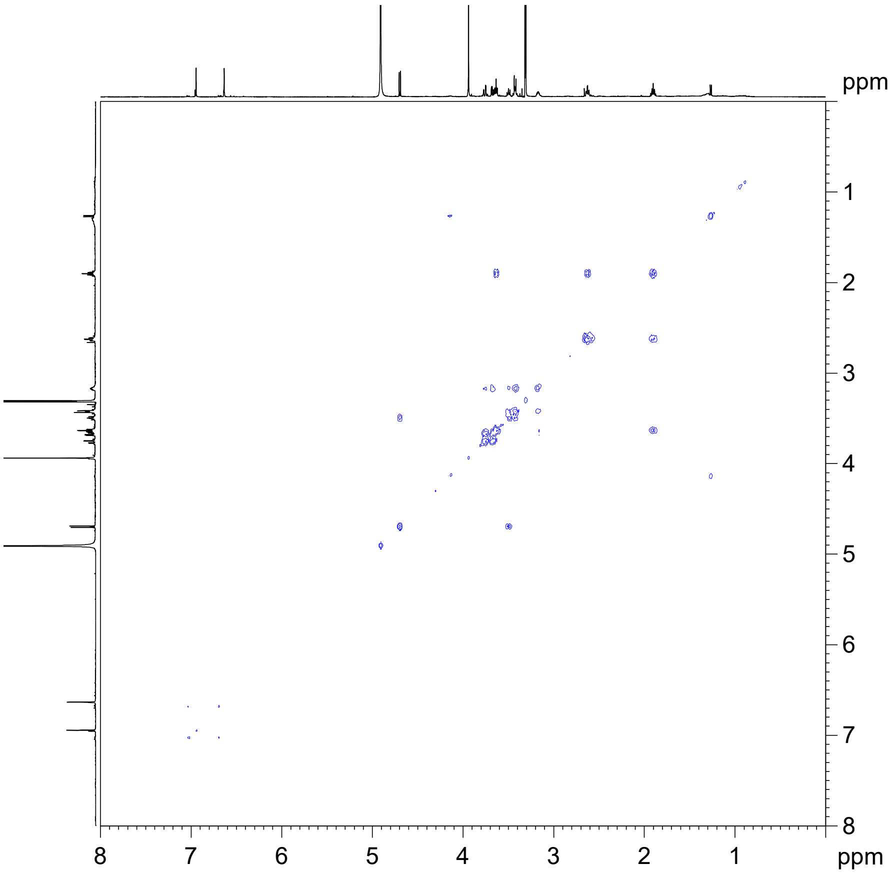

Figure S23. ^{1}H -^{1}H COSY spectrum (500 MHz, CD_3OD) of **4**

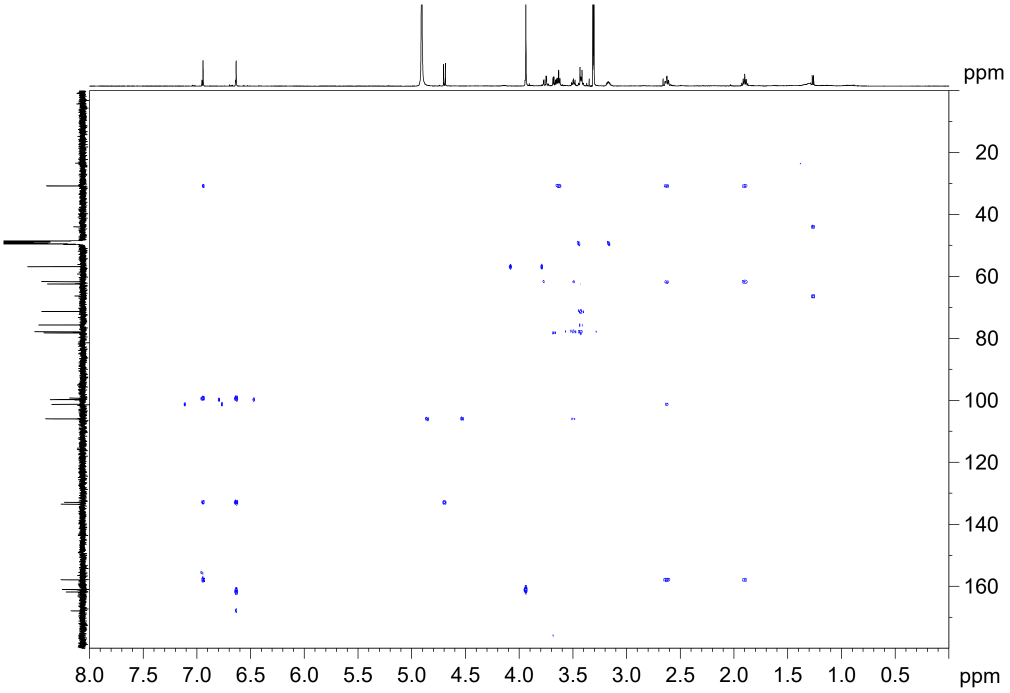

Figure S24. HMBC spectrum (500 MHz, CD_3OD) of **4**

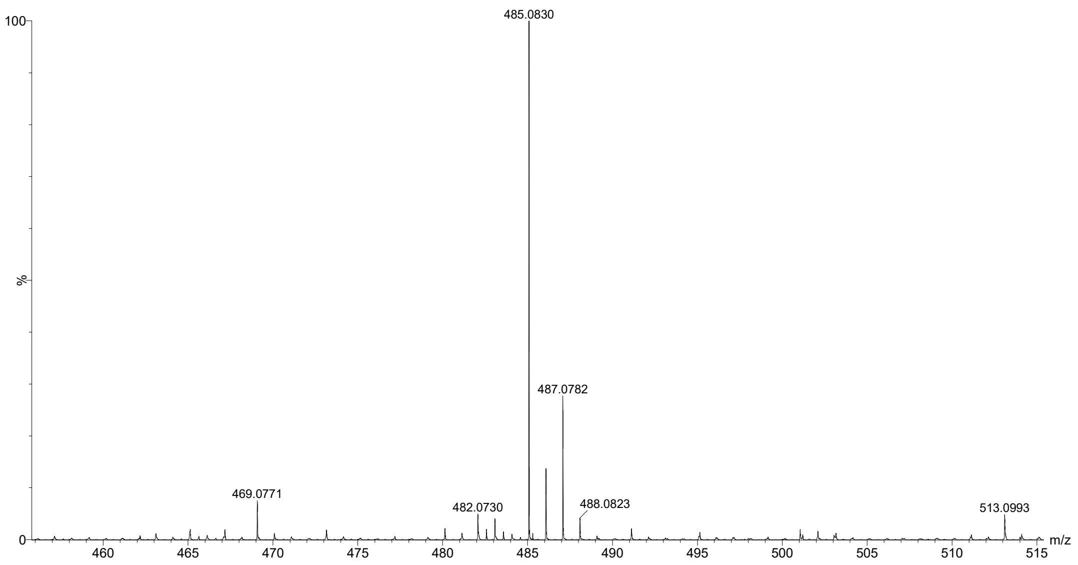

Figure S25. HRESIMS spectrum for compound **5**

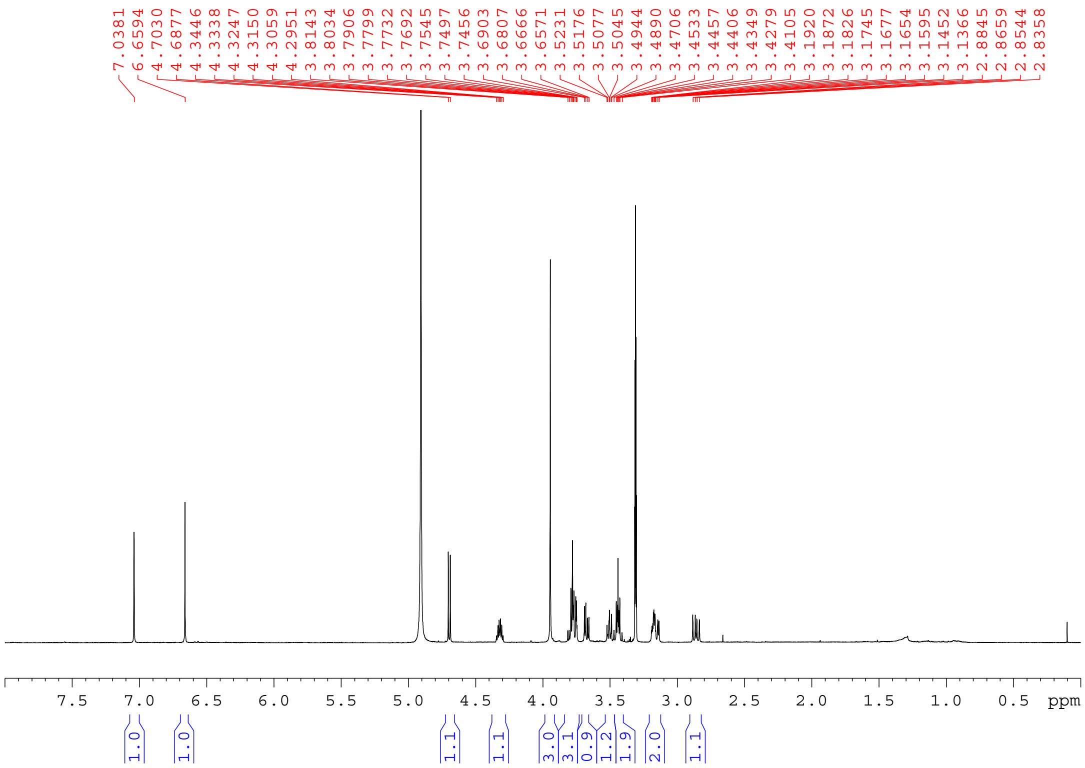

Figure S26. ¹H NMR spectrum (500 MHz, CD3OD) of **5**

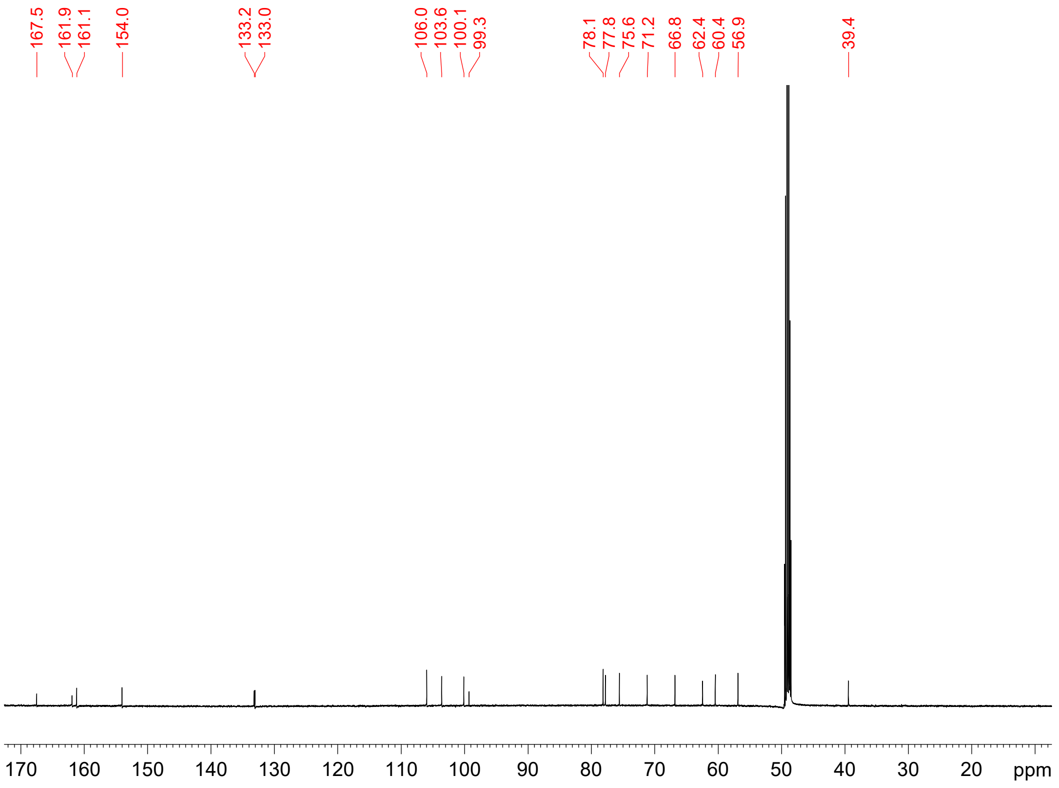

Figure S27. ^{13}C NMR spectrum (125 MHz, CD_3OD) of **5**

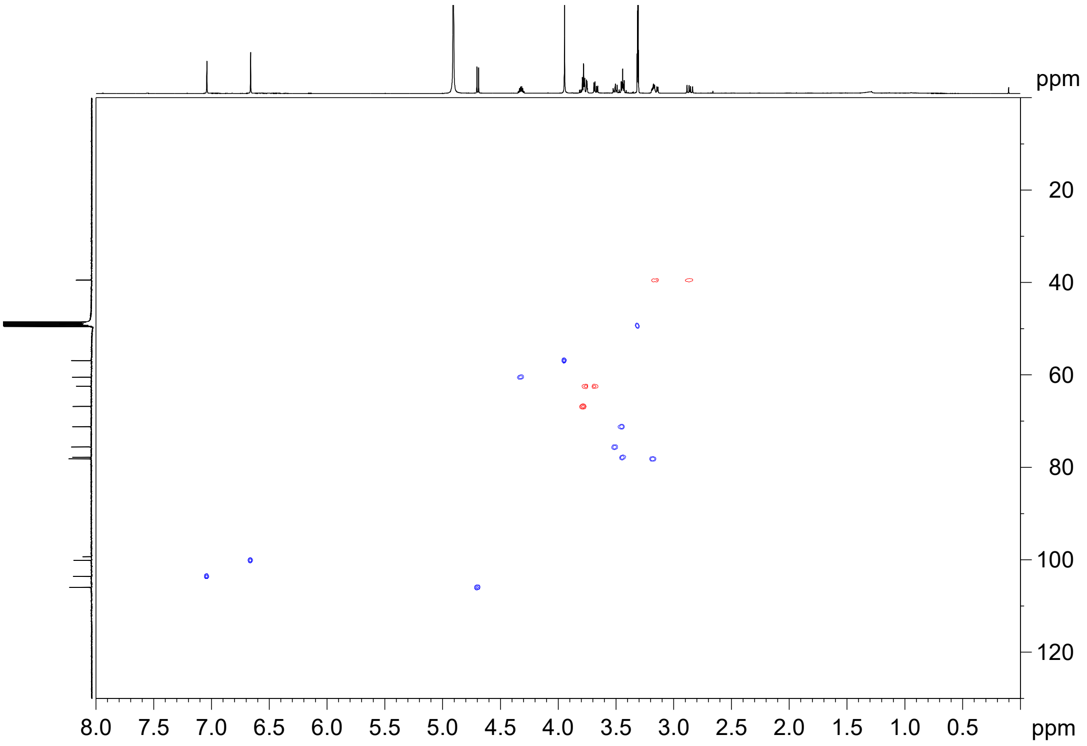

Figure S28. HSQC spectrum (500 MHz, CD_3OD) of **5**

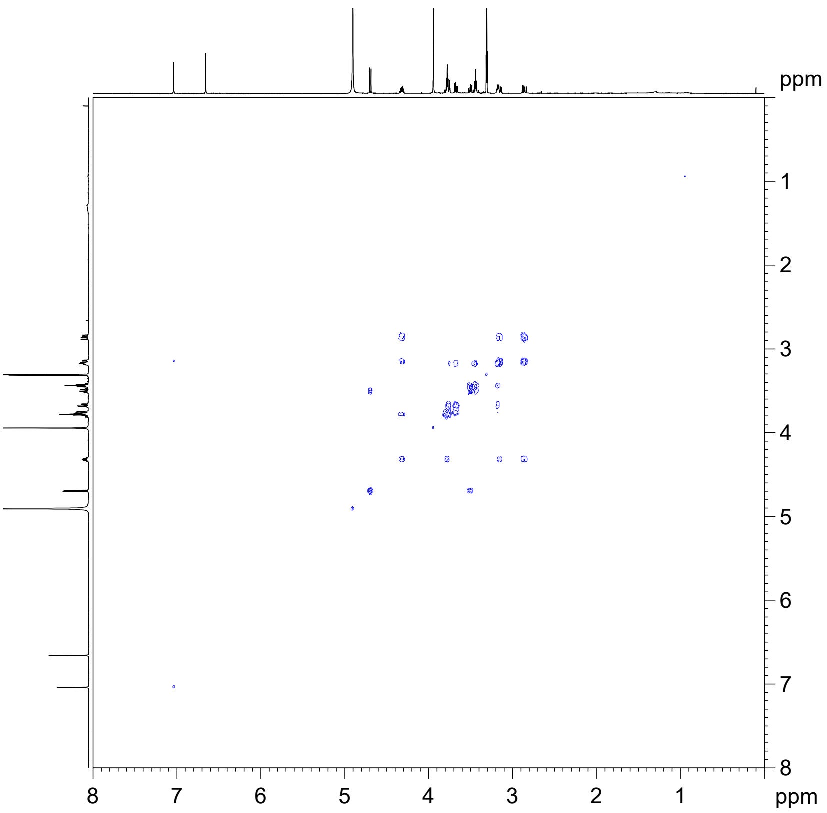

Figure S29. ^{1}H -^{1}H COSY spectrum (500 MHz, CD_3OD) of **5**

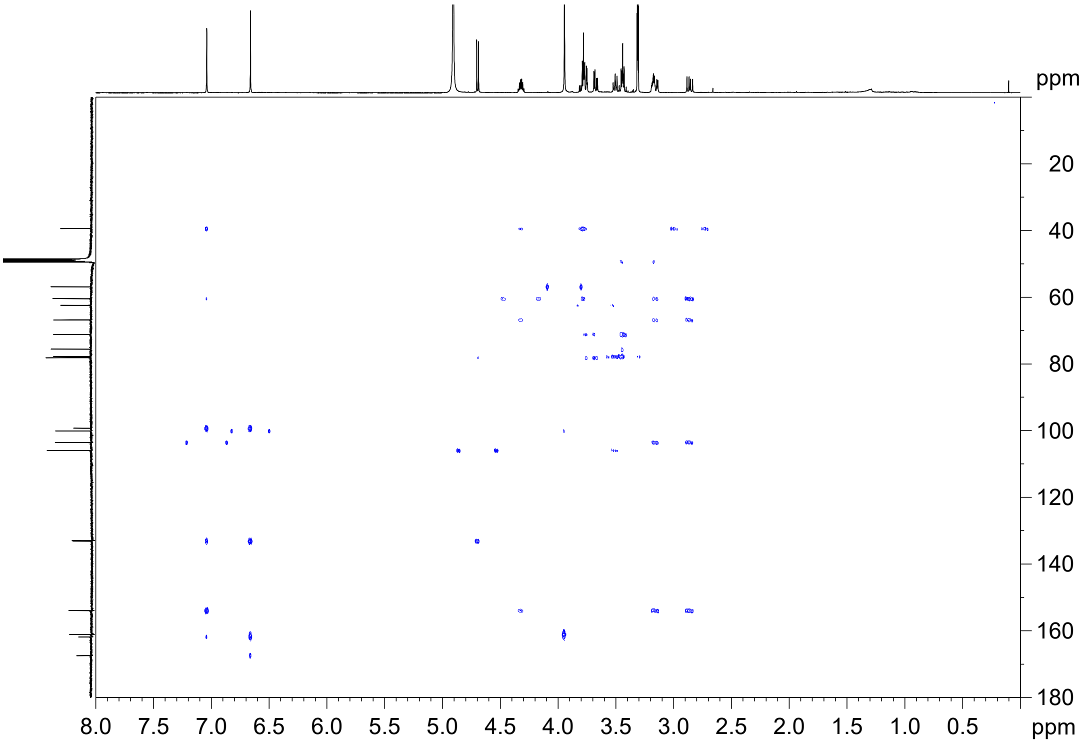

Figure S30. HMBC spectrum (500 MHz, CD_3OD) of **5**

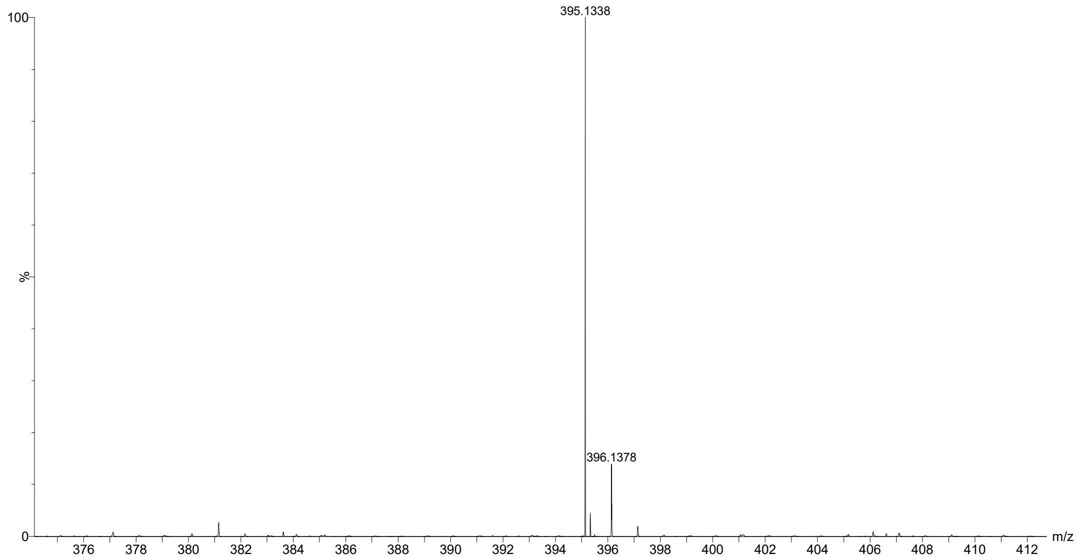

Figure S31. HRESIMS spectrum for compound **6**

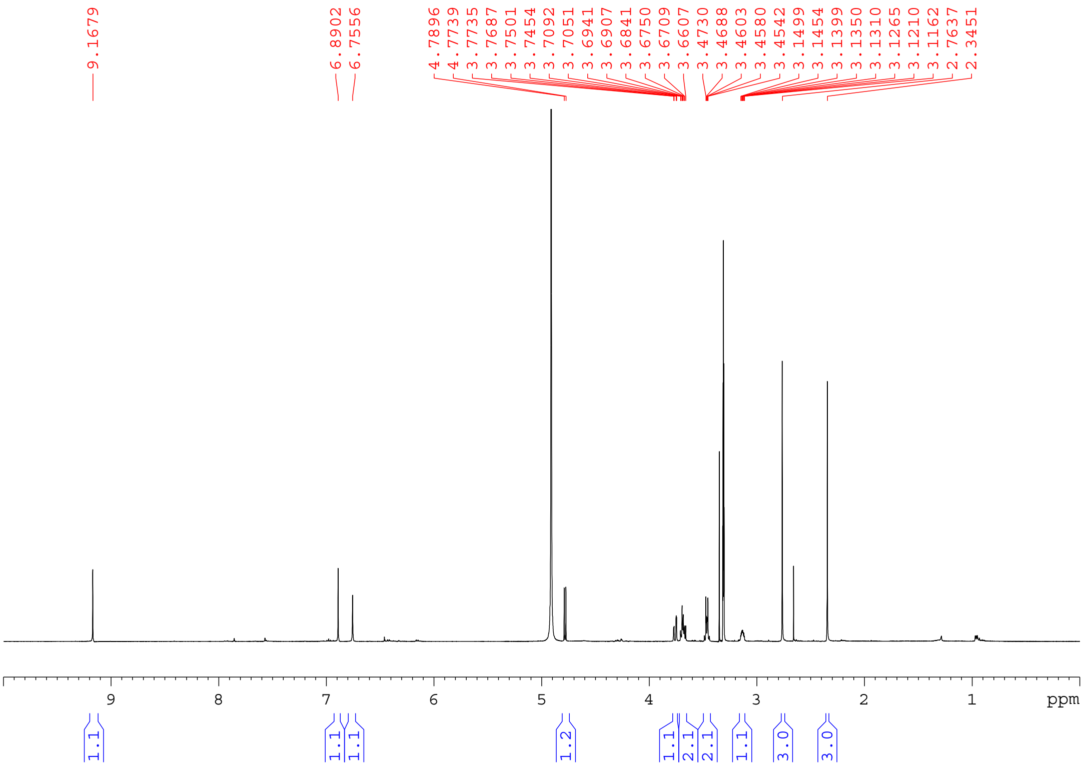

Figure S32. ^{1}H NMR spectrum (500 MHz, CD_3OD) of **6**

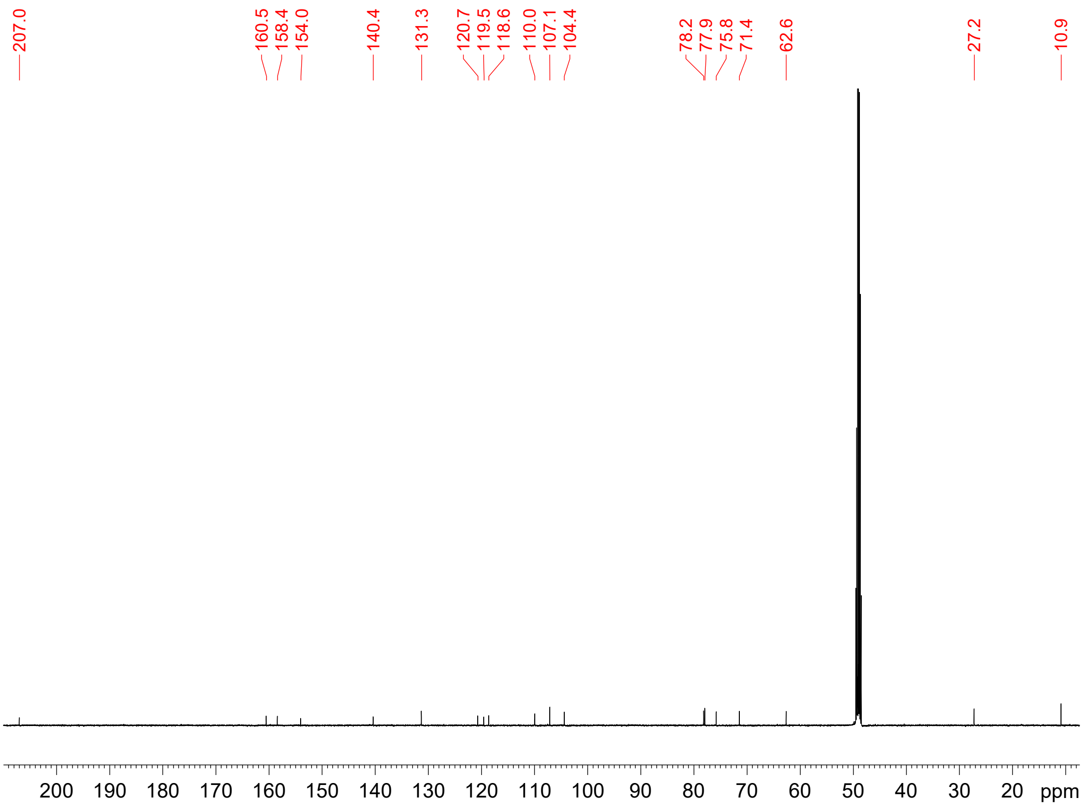

Figure S33. ^{13}C NMR spectrum (125 MHz, CD_3OD) of **6**

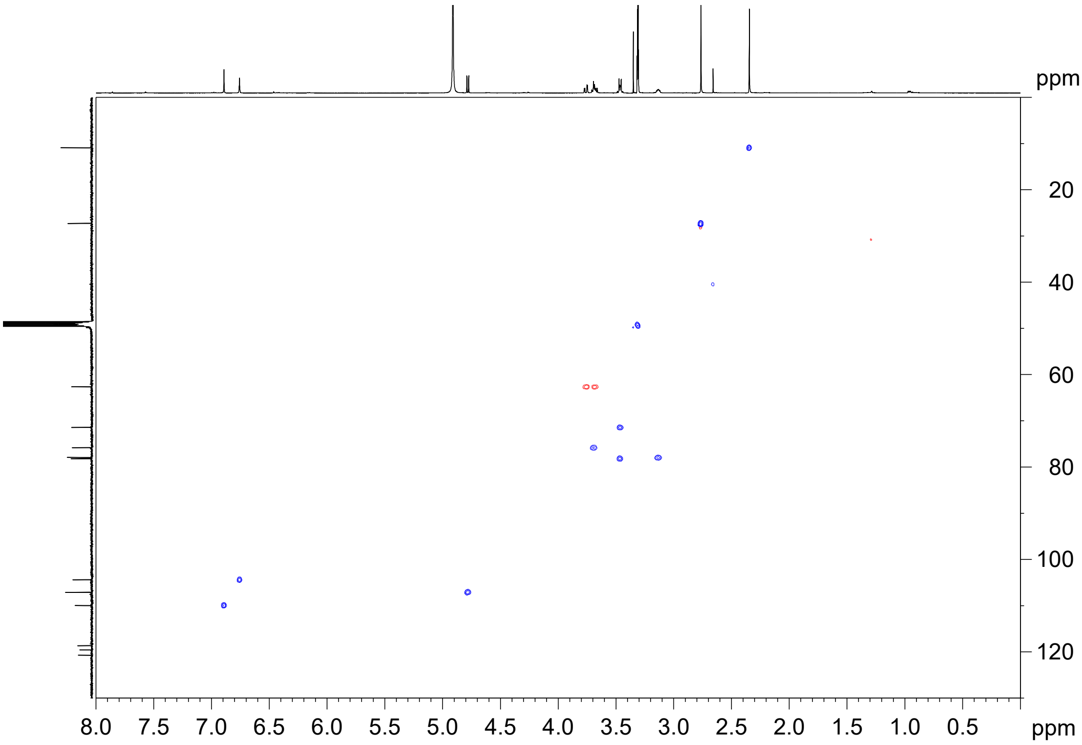

Figure S34. HSQC spectrum (500 MHz, CD_3OD) of **6**

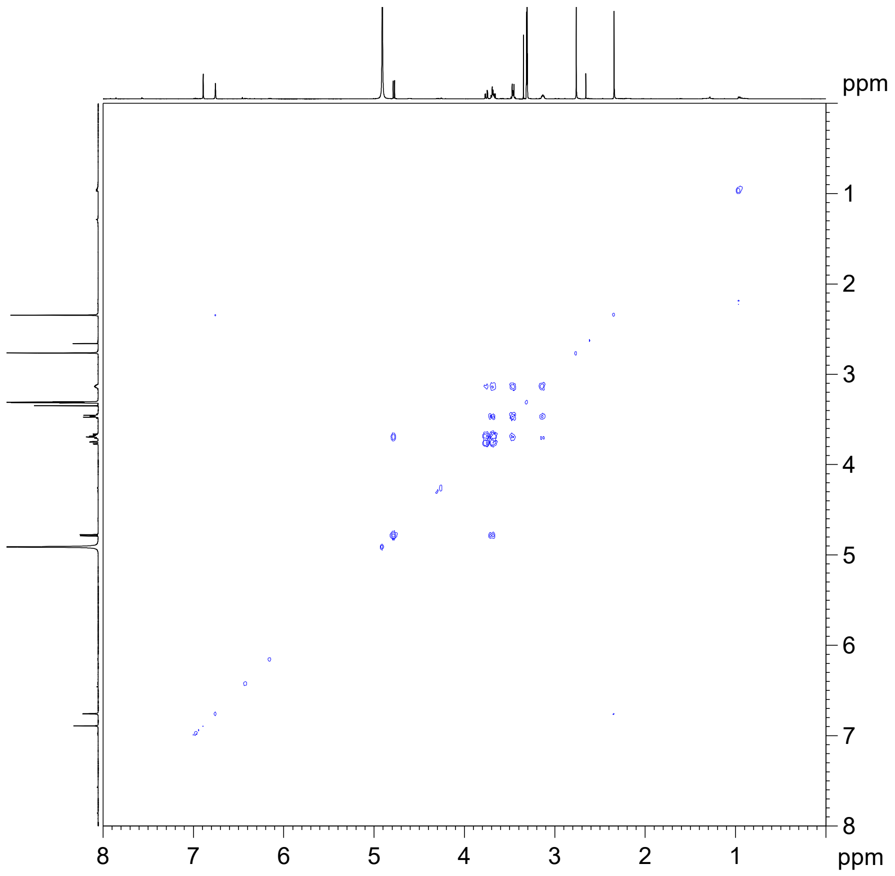

Figure S35. ^{1}H -^{1}H COSY spectrum (500 MHz, CD_3OD) of **6**

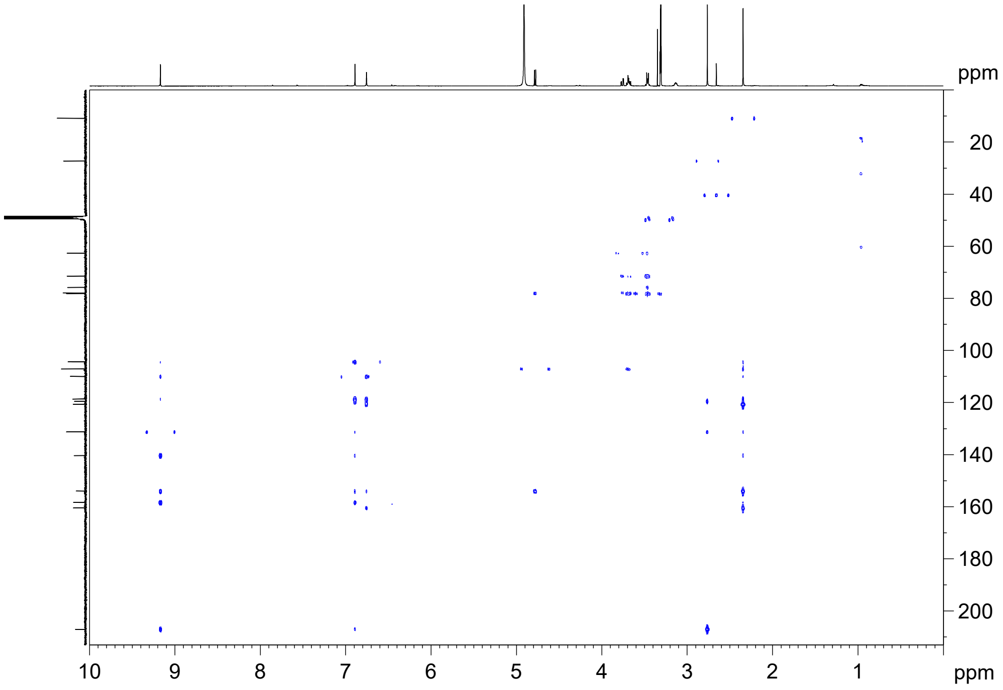

Figure S36. HMBC spectrum (500 MHz, CD_3OD) of **6**

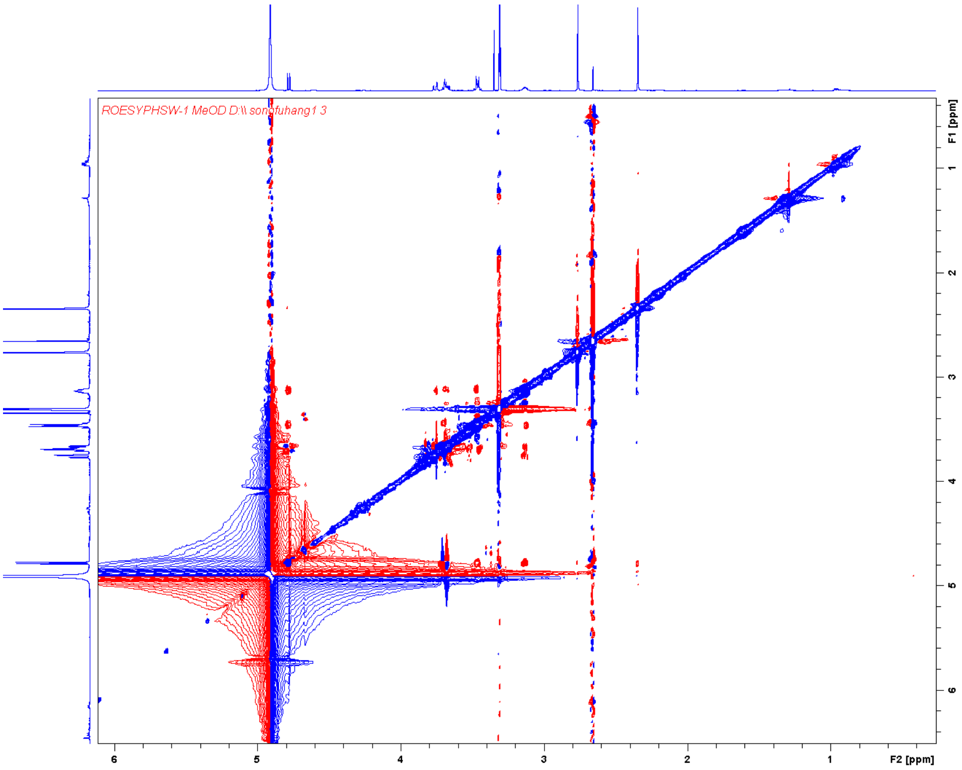

Figure S37. ROESY spectrum (500 MHz, CD_3OD) of **6**

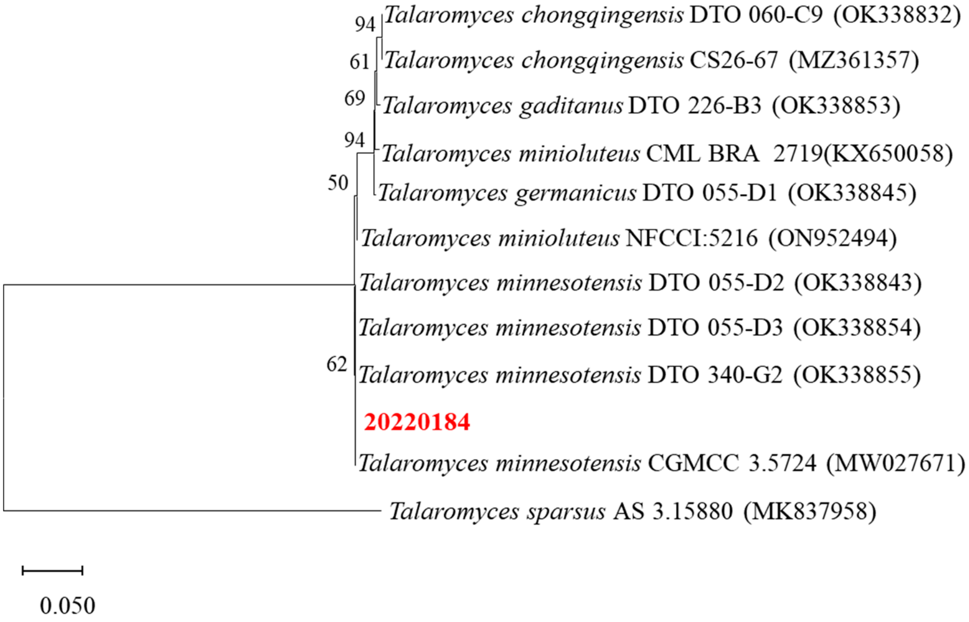

Figure S38. Neighbor-joining phylogenetic tree of BTBU20220184 and its most related type strains based on internal transcribed spacer region (ITS) from NCBI ITS database. Numbers at nodes indicate levels of bootstrap support (%) based on a neighbor joining analysis of 1,000 resampled datasets; only values >50 % are given. NCBI accession numbers are given in parentheses. Bar 0.005 nucleotide substitutions per site.
